# Aqueous Persistent Noncovalent Ion-Pair Cooperative Coupling in a Ruthenium Cobaltabis(dicarbollide) System as a Highly Efficient Photoredox Oxidation Catalyst

**DOI:** 10.1021/acs.inorgchem.1c00751

**Published:** 2021-06-07

**Authors:** Isabel Guerrero, Clara Viñas, Xavier Fontrodona, Isabel Romero, Francesc Teixidor

**Affiliations:** † Institut de Ciencia de Materials de Barcelona, Consejo Superior de Investigaciones Científicas, Campus UAB, E-08193 Bellaterra, Spain; ‡ Departament de Química and Serveis Tècnics de Recerca, Universitat de Girona, c/m Aurèlia Campmany 69, E-17003 Girona, Spain

## Abstract

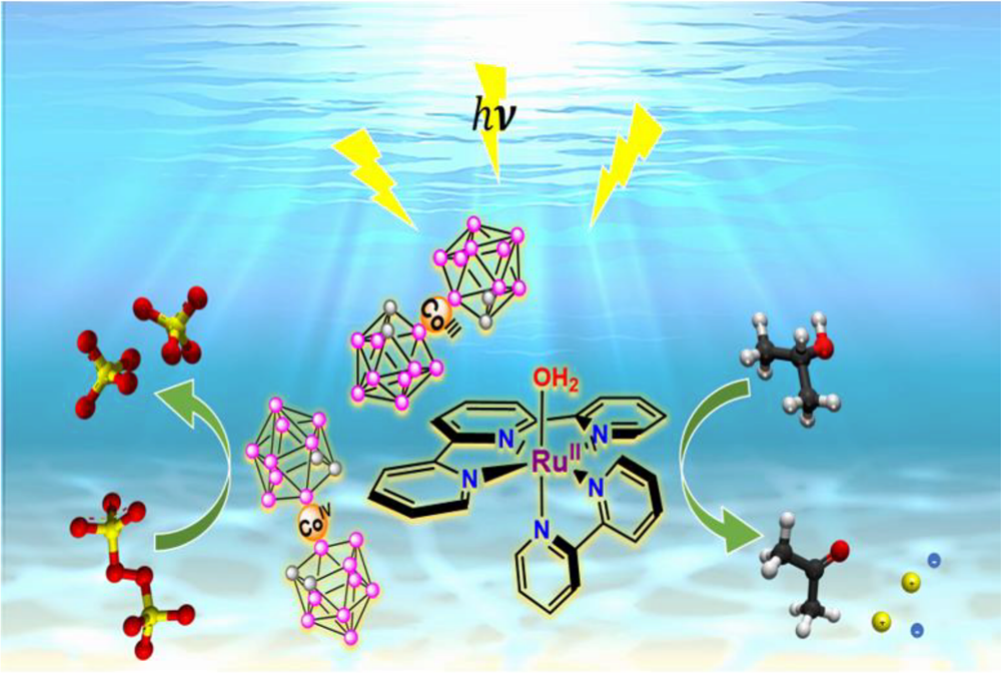

An original cooperative photoredox catalytic system, [Ru^II^(trpy)(bpy)(H_2_O)][3,3′-Co(1,2-C_2_B_9_H_11_)_2_]_2_ (**C4**; trpy = terpyridine and bpy = bipyridine), has been synthesized. In this system, the photoredox metallacarborane catalyst [3,3′-Co(1,2-C_2_B_9_H_11_)_2_]^−^ (**[1]^−^**) and the oxidation catalyst [Ru^II^(trpy)(bpy)(H_2_O)]^2+^ (**C2′**) are linked by noncovalent interactions and not through covalent bonds. The noncovalent interactions to a large degree persist even after water dissolution. This represents a step ahead in cooperativity avoiding costly covalent bonding. Recrystallization of **C4** in acetonitrile leads to the substitution of water by the acetonitrile ligand and the formation of complex [Ru^II^(trpy)(bpy)(CH_3_CN)][3,3′-Co(1,2-C_2_B_9_H_11_)_2_]_2_ (**C5**), structurally characterized. A significant electronic coupling between **C2′** and **[1]^−^** was first sensed in electrochemical studies in water. The Co^IV/III^ redox couple in water differed by 170 mV when **[1]^−^** had Na^+^ as a cation versus when the ruthenium complex was the cation. This cooperative system leads to an efficient catalyst for the photooxidation of alcohols in water, through a proton-coupled electron-transfer process. We have highlighted the capacity of **C4** to perform as an excellent cooperative photoredox catalyst in the photooxidation of alcohols in water at room temperature under UV irradiation, using 0.005 mol % catalyst. A high turnover number (TON = 20000) has been observed. The hybrid system **C4** displays a better catalytic performance than the separated mixtures of **C2′** and **Na[1]**, with the same concentrations and ratios of Ru/Co, proving the history relevance of the photocatalyst. Cooperative systems with this type of interaction have not been described and represent a step forward in getting cooperativity avoiding costly covalent bonding. A possible mechanism has been proposed.

## Abstract 2

A cooperative ion-pair photoredox system linked by noncovalent interactions and formed by cobaltabis(dicarbollide) as the light collector and a ruthenium aqua complex as the electron-transfer agent has proven to be an efficient photoredox oxidation catalyst in water through a proton-coupled electron transfer.

## Introduction

The development of new photocatalytic methods for organic transformations is an important challenge given the significant environmental and economic impact that this entails. Solar energy combined with water are widely considered to be good alternative energy sources for the development of nonfossil-based fuels.^1,2^ Great effort has been dedicated to the catalysis activation of organic molecules by light.^3−5^ The latter can be considered to be a good reagent for environmentally friendly, green chemical synthesis; unlike many conventional reagents, light is an abundant energy form for driving chemical reactions in a sustainable future.

The activation of molecules by light depends on the ability of metal complexes and organic dyes to participate in electron-transfer (ET) processes with organic molecules upon excitation by light.^6,7^ The octahedral compound [Ru(bpy)_3_]^2+^ (bpy = bipyridine)^8^ has been one of the most studied photoredox catalysts in the literature; it participates in ET by an outer-sphere mechanism that supposes no significant structural modification upon ET. The challenge is to generate charge separation by ET and maintain it by avoiding regeneration of the initial reagent.

Cooperative photoredox catalysis supposes a new progress in this field and refers to a first catalyst that is photochemically active, while the second is redox-active in the absence of light.^9^ This strategy allows multielectron photoredox catalysis with the help of a second redox catalyst, avoiding the single electron transfer (SET) imposed by the photoredox catalyst. The union in one single complex of a photoredox catalyst and a transition-metal catalyst could result in higher efficiency and may lead to new selective light-induced organic transformations.

Currently, systems that are used for the light-induced oxidation of substrates are based on chromophore–catalyst dyads of ruthenium polypyridyl compounds,^10−12^ where one compound acts as the light-harvesting antenna and the second metal complex is used as catalyst, to activate a water molecule or an organic substrate. In these cases, the problem is the charge trapping between the two moieties influenced by a bridging ligand that connects both parts, leading to a reduction of the catalytic activity observed in some systems. Moreover, the synthesis of these systems can be laborious and expensive.

Oxidized raw materials are needed as bulk chemicals in different fields, including polymer and fine chemicals, among others.^13^ So, the selective oxidation of alcohols is an essential process in organic chemistry^14−17^ because this oxidation can be used to produce aldehydes, acids, ketones, etc.

It is also relevant in hydrogen-based energy technologies because this reaction involves a two-electron proton-coupled process that could be recombined on a cathode for hydrogen production.^18,19^

It is well-known that carboranes interact with light^20^ and that they have been studied as catalysts in different processes.^21^ The use of carboranes in supramolecular chemistry is a field to be explored for their particular characteristics.^22−25^ The metallacarborane sandwich compound [3,3′-Co(1,2-C_2_B_9_H_11_)_2_]^−^ (**[1]^−^**) has many possibilities of forming hydrogen bonds, e.g., C_c_–H···O or C_c_–H···X (X = halogen) as well as dihydrogen bonding C_c_–H···H–B and B–H···H–N (C_c_ stands for the cluster C atoms).^26−28^ It is well-known that **[1]^−^** is highly stable in water but at low concentration forms aggregates (vesicles and micelles).^29,30^ Dihydrogen interactions participate in the self-assembling formation.^31^ These supramolecular interactions appear to be significant in the processes of ET and therefore in the performance and efficiency of photocatalytic systems.^32^ Recently, we have shown that cobaltabis(dicarbollide), **[1]^−^**, and its chloro derivatives, acting as both catalyst and photosensitizer, are highly efficient in the photooxidation of alcohols in water, through SET processes.^33^ A high performance of **Na[1]** in the photooxidation of alcohols is observed, using a catalyst load of 0.01 mol % and reaching TON = 10000, in some cases.^33^ We have also supported the cobaltabis(dicarbollide) catalyst on silica-coated magnetite nanoparticles.^34^ This system has proven to be a green and sustainable heterogeneous catalytic system, highly efficient and easily reusable for the photooxidation of alcohols in water. In general, the SET processes carry out the transformation of a limited number of substrates, and in some cases undesirable byproducts are generated. As we have indicated, the results offered by **[1]^−^** in SET have been excellent, taking into account the low catalyst loading, the selectivity, and the high degree of conversion. However, we wanted to know more about their behavior, whether cooperative action offered any advantage, whether a cooperative effect really existed, and whether the noncovalent interactions demonstrated in water had a history. To this end, the studies with alcohol oxidations already studied only with **[1]^−^** would allow us to prove the features that have not been demonstrated or found in other related systems. Once these points are known and demonstrated, we will be in the position to develop unprecedented systems.

As mentioned above, **[1]^−^** can form ion-pair complexes through hydrogen and dihydrogen interactions.^31^ Then, the design of a new hybrid system where cobaltabis(dicarbollide) can be linked by noncovalent interactions to a redox-active oxidation catalyst would lead to the formation of a stable and efficient cooperative photoredox catalyst.

**Scheme 1:**
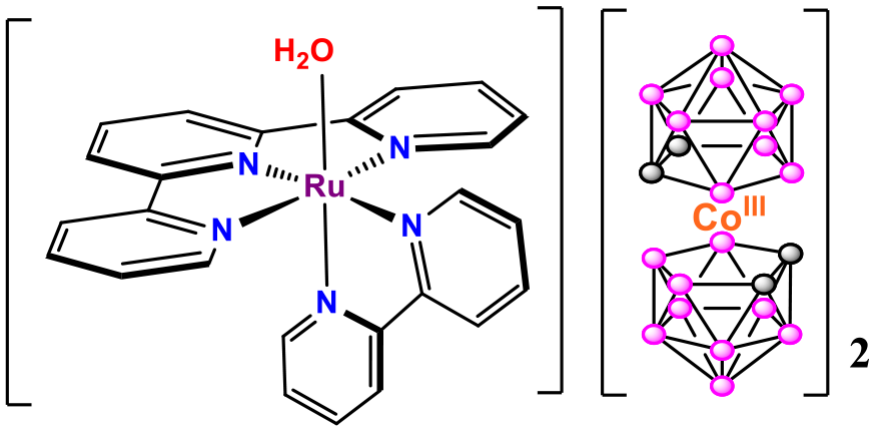
Schematic Representation of the Ruthenium Cobaltabis(dicarbollide) Complex **C4**

With all this in mind, in this work we present the synthesis of an air-stable ruthenium cobaltabis(dicarbollide) compound, [Ru^II^(trpy)(bpy)(H_2_O)][3,3′-Co(1,2-C_2_B_9_H_11_)_2_]_2_ (**C4**, where trpy = terpyridine and bpy = bipyridine; Scheme 1), where the [Ru-OH_2_] cation belongs to the family of redox oxidation catalysts,^35,36^ together with their spectroscopic and electrochemical characterization. The recrystallization of **C4** in acetonitrile leads to formation of the complex [Ru^II^(trpy)(bpy)(CH_3_CN)][3,3′-Co(1,2-C_2_B_9_H_11_)_2_]_2_ (**C5**), which has been structurally characterized. **C4** has been tested as a cooperative photoredox catalyst in the oxidation of aromatic and aliphatic alcohols in water showing high performance, using very low catalyst loads.

## Results and Discussion

### Synthetic Strategy, Structure, and Redox Characterization

**Scheme 2:**
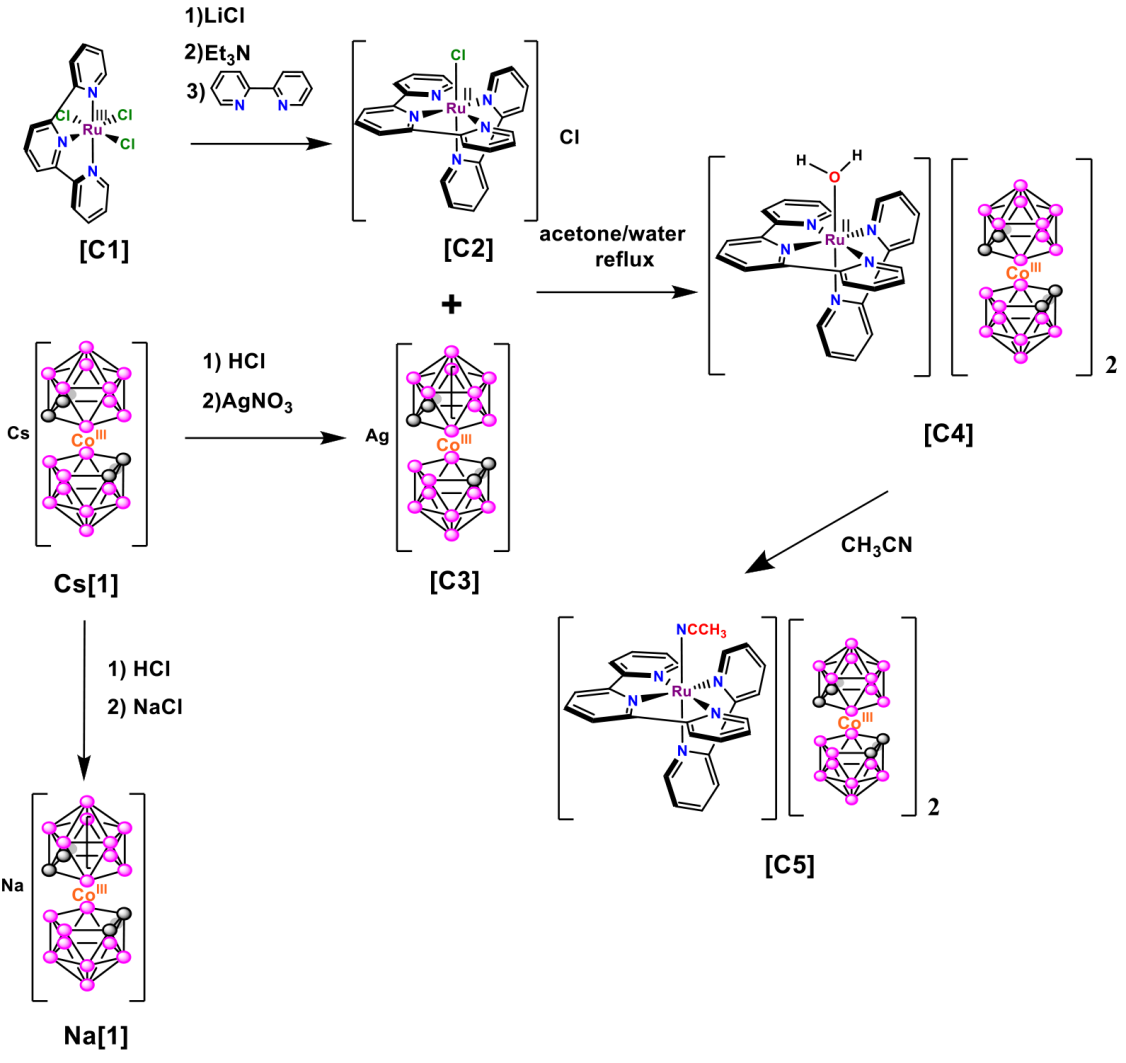
Synthetic Strategy for the Preparation of Complexes **C4** and **C5**

The synthetic process used for preparation of the compounds is outlined in Scheme 2. The aqua complex **C4** is obtained by dissolving the chloridoruthenium(II) complex [Ru^II^(trpy)(bpy)(Cl)]Cl (**C2**), obtained following the method described in the literature,^37^ in a mixture of water/acetone (1:1) in the presence of **Ag[1]**, **C3**, under reflux. This latter compound was prepared by a cationic exchange resin from **Cs[1]**, as described in the literature.^38^ After filtration of AgCl, the complex was isolated. The recrystallization of **C4** in acetonitrile led to the substitution of water by the acetonitrile ligand, and then the formation of complex **C5** took place. It is worth mentioning that, to the best of our knowledge, this is the first example of a molecular aqua ruthenium(II) complex containing two cobaltabis(dicarbollide) anions as counterions.

Suitable single crystals of **C5** were grown by the recrystallization of **C4** in acetonitrile. All attempts to obtain crystals of **C4** in water or noncoordinating solvents were unsuccesful.

**Figure 1:**
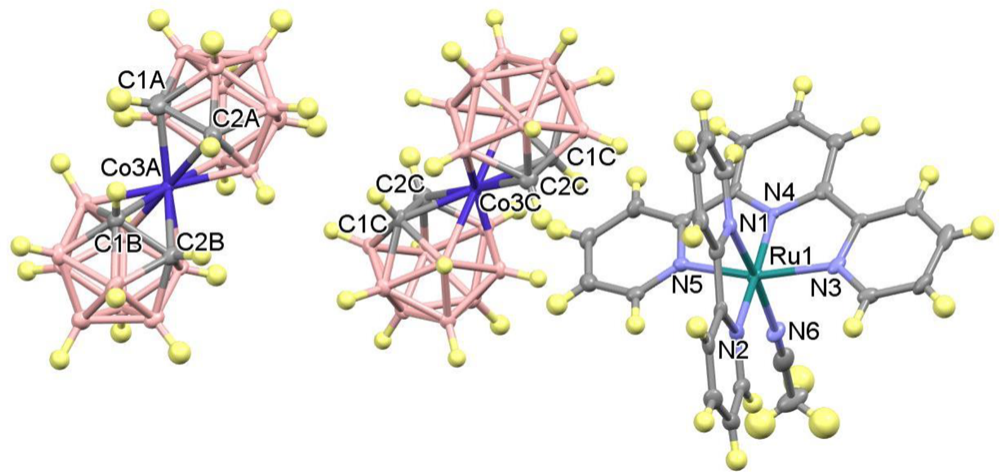
Crystal structure of complex **C5**.

X-ray diffraction analysis was used to solve the crystal structure of complex **C5** (Figure 1). The main crystallographic data, together with selected bond distances and angles, are reported in Tables S1 and S2. The cationic moiety of compound **C5** is formed by a ruthenium(II) complex, where the Ru center displays an octahedral distorted type of coordination, in which the trpy ligand is bonded to the Ru^II^ cation in a meridional manner and the bpy ligand is coordinated in a bidentate fashion. The sixth coordination site is occupied by the monodentate acetonitrile ligand. All bond distances and angles are within the expected values for these types of complexes.^39−41^ For instance, it is interesting to note that the Ru–N2 bond length in the bpy ligand, where the N2 atom is placed trans to the pyridyl N4 atom of trpy, is longer (2.069 Å) than the analogous distance Ru–N1 (2.033 Å), where the N1 atom is trans to the N6 atom of the acetonitrile ligand. This evidence highlights the stronger trans influence of the pyridine ring with respect to the acetonitrile ligand. The N3–Ru–N4 and N4–Ru–N5 angles are 79.88° and 79.13°, respectively, less than the 90° expected for an ideal octahedral geometry. This may be due to the geometry of the trpy ligand, which defines the equatorial plane of the structure; as a consequence, the other two equatorial angles, N3–Ru–N2 and N2–Ru–N5, are larger than 90°. The anionic moiety is formed by two anionic metallabis(dicarbollide) units, which display different rotamers: one cisoid and the other transoid. The C1A–C2A (1.661 Å) and C1B–C2B (1.642 Å) bond lengths are different in the cisoid anion, while the C1C–C2C bond lengths are similar (1.649 Å) in the transoid rotamer anion. The packing in Figure S1b displays the arrangement adopted between the metallacarborane anions and cationic moieties.

The IR spectrum of complex **C4** shows vibrations around 2900 cm^–1^ that can be assigned to the ν_C–H_ stretching modes for the aromatic rings, while the bands around 3030 cm^–1^ correspond to the ν_C–H_ stretching of the C_c_–H bonds in the different rotamers. A band over 3500 cm^–1^ can be seen that corresponds to the ν_O–H_ stretching of the aqua ligand. We have also observed a significant vibration around 2530 cm^–1^ that corresponds to the ν_B–H_ stretching mode for the B–H bond (Figure S2). The 1D and 2D ^1^H NMR spectra of complexes **C3** and **C4** were recorded in acetone-*d*_6_ and are displayed in Figures S3 and S4. The ^1^H NMR spectrum of **C4** shows two sets of signals (Figure S4a): one in the aromatic region corresponding to the protons of the bipyridine and terpyridine ligands of the [Ru−OH_2_]^2+^ cation and the second set in the aliphatic region that can be assigned to the C_c_–H of the cobaltacarborane anions, whose resonances appear near δ = 3.98 ppm. The ^1^H{^11^B} NMR spectrum exhibits the resonances of H atoms bonded to B atoms over a wide chemical-shift range in the region from δ = 1.50 to 3.5 ppm. These resonances appear as broad bands (Figure S4b).

The ^11^B{^1^H} NMR resonances featured the typical pattern of the pristine cobaltabis(dicarbollide) cluster in the range from δ = 15 to −22 ppm (Figure S4c).^42^

**Figure 2:**
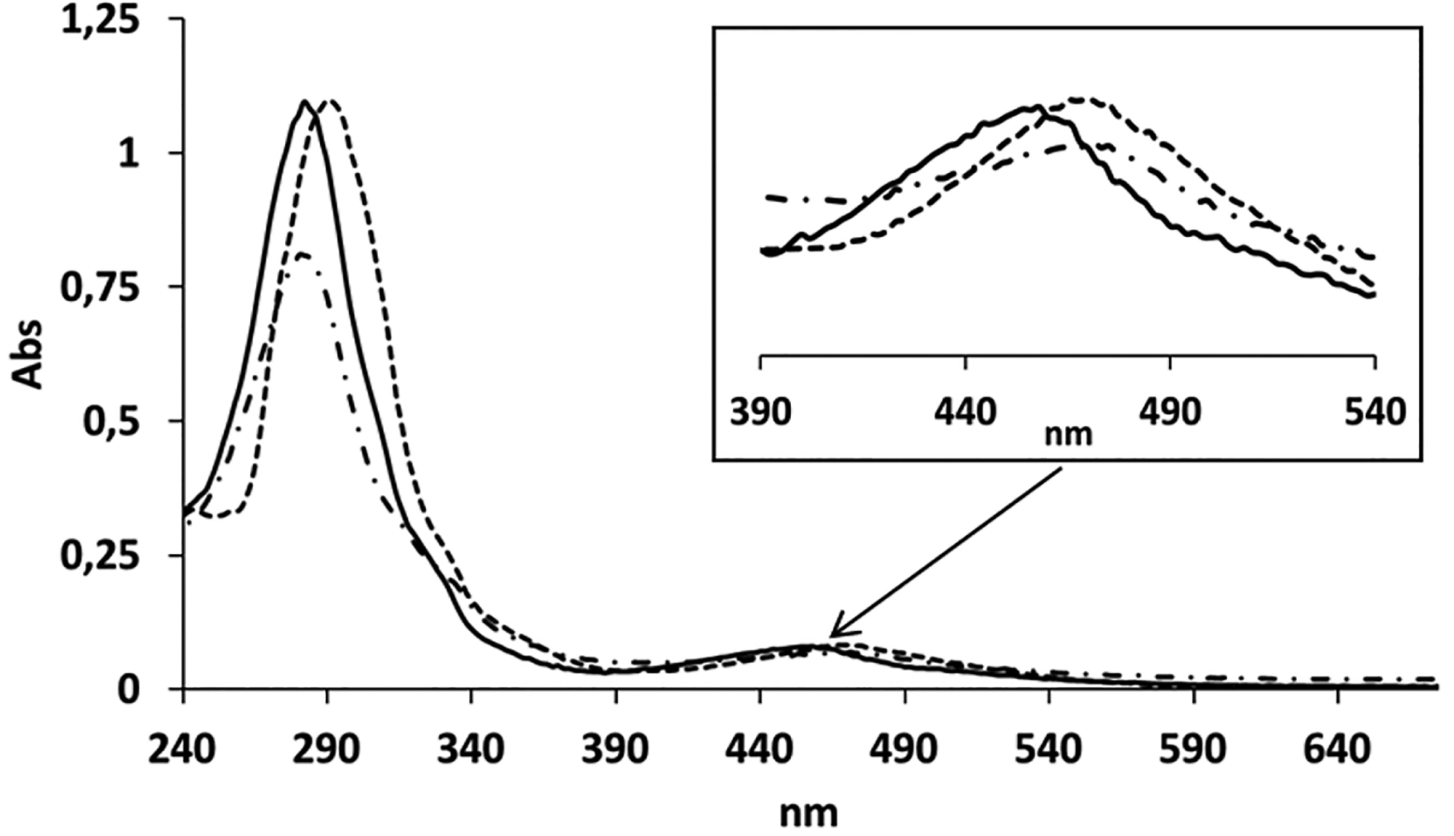
UV–vis spectra of complex **C4** in CH_2_Cl_2_ (dotted line), phosphate buffer at 7.02 pH (dash-dotted line), and CH_3_CN (solid line).

The UV–vis spectra of **Ag[1]**, **C3**, and **C2′**, together with **C4**, are displayed in Figure S5a,b. The UV–vis spectrum of **Ag[1]**, **C3** (Figure S5a), shows one strong absorption band at 286 nm and others with less intensity at 210, 333, and 450 nm, in agreement with the literature.^43,44^ The spectrum of the aqua complex **C2′** (Figure S5b) shows ligand-based π–π* bands and dπ(Ru)−π*(L) metal-to-ligand charge-transfer (MLCT) transitions, as is usual for these complexes.^45^ In Figures S5b and 2 is shown the UV–visible spectrum corresponding to complex **C4**, which exhibits the ligand-based π–π* bands of the cationic part below 350 nm that are partially eclipsed by strong absorptions corresponding to the cobaltabis(dicarbollide) anionic moiety. Above 350 nm, the spectra show less intense bands that correspond to dπ(Ru)−π*(L) MLCT transitions. Figure 2 displays the UV–vis spectra of **C4** in CH_2_Cl_2_, phosphate buffer, and CH_3_CN. It is worth mentioning the shift to higher energy absorptions observed for **C4** in acetonitrile with regard to the same complex in water or dichloromethane. This is expected because of substitution of the aqua ligand by acetonitrile; this ligand shows a higher π-acceptor capacity with respect to the aqua ligand, which provokes stabilization of the dπ(Ru) donor orbitals.

The electrochemical behavior of complexes **C3**, **Na[1]**, and **C4** have been studied by means of cyclic voltammetry (CV) and differential pulse voltammetry (DPV) (Figures S6–S8). The CV curve of **C3** in acetonitrile shows two reversible one-electron-redox processes at *E*_1/2_ = −1.69 and 1.27 V versus Ag/AgCl as reference electrode, which can be assigned to Co^III^/Co^II^ and Co^IV^/Co^III^, respectively (Figure S6a). The CV curve of the aqua complex **C4** in dichloromethane exhibits different redox processes because of the Ru and Co ions. Two are almost reversible monoelectronic Co^III^Ru^III^/Co^III^Ru^II^ and Co^III^Ru^IV^/Co^III^Ru^III^ redox waves at *E*_1/2_ around 0.68 and 0.96 V versus Ag and two monoelectronic Co^III^Ru^II^/Co^II^Ru^II^ and Co^IV^Ru^IV^/Co^III^Ru^IV^ redox waves at *E*_1/2_ = −1.57 and 1.38 V Versus Ag/AgCl, respectively (Figure S6b).

**Figure 3:**
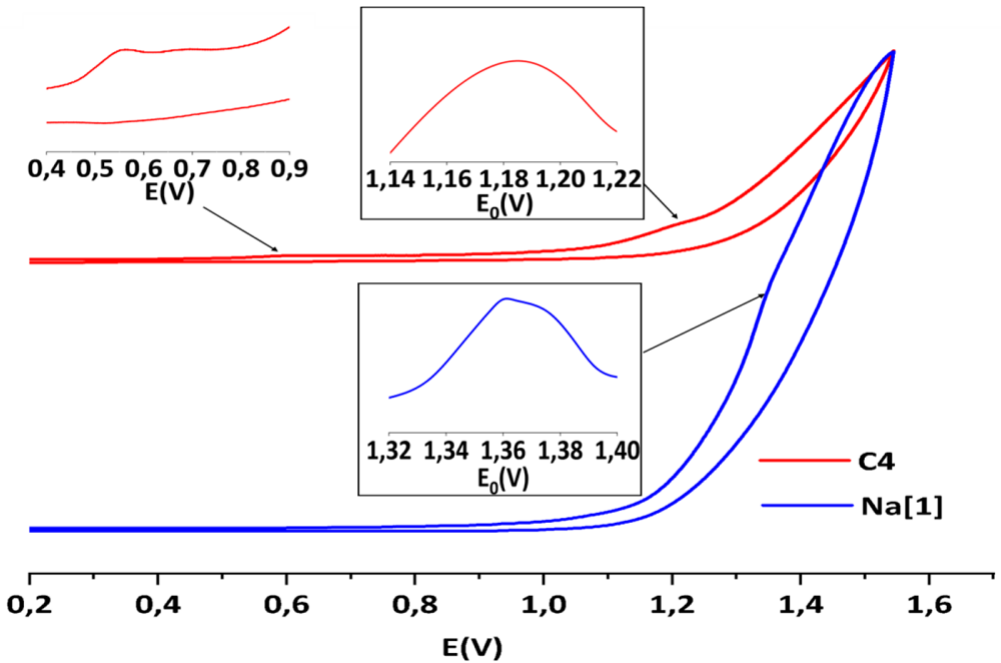
CV for complexes **C4** (red line) and **Na[1]** (blue line) registered in a phosphate buffer (pH = 7.12) versus Ag/AgCl. The right red and blue insets show d*I*/d*E* to better appreciate the position of the couple Co^IV^/Co^III^. Further proof of this will be shown later when dealing with the **C4** precedents that we will henceforth call the **C4** history.

**Figure 4:**
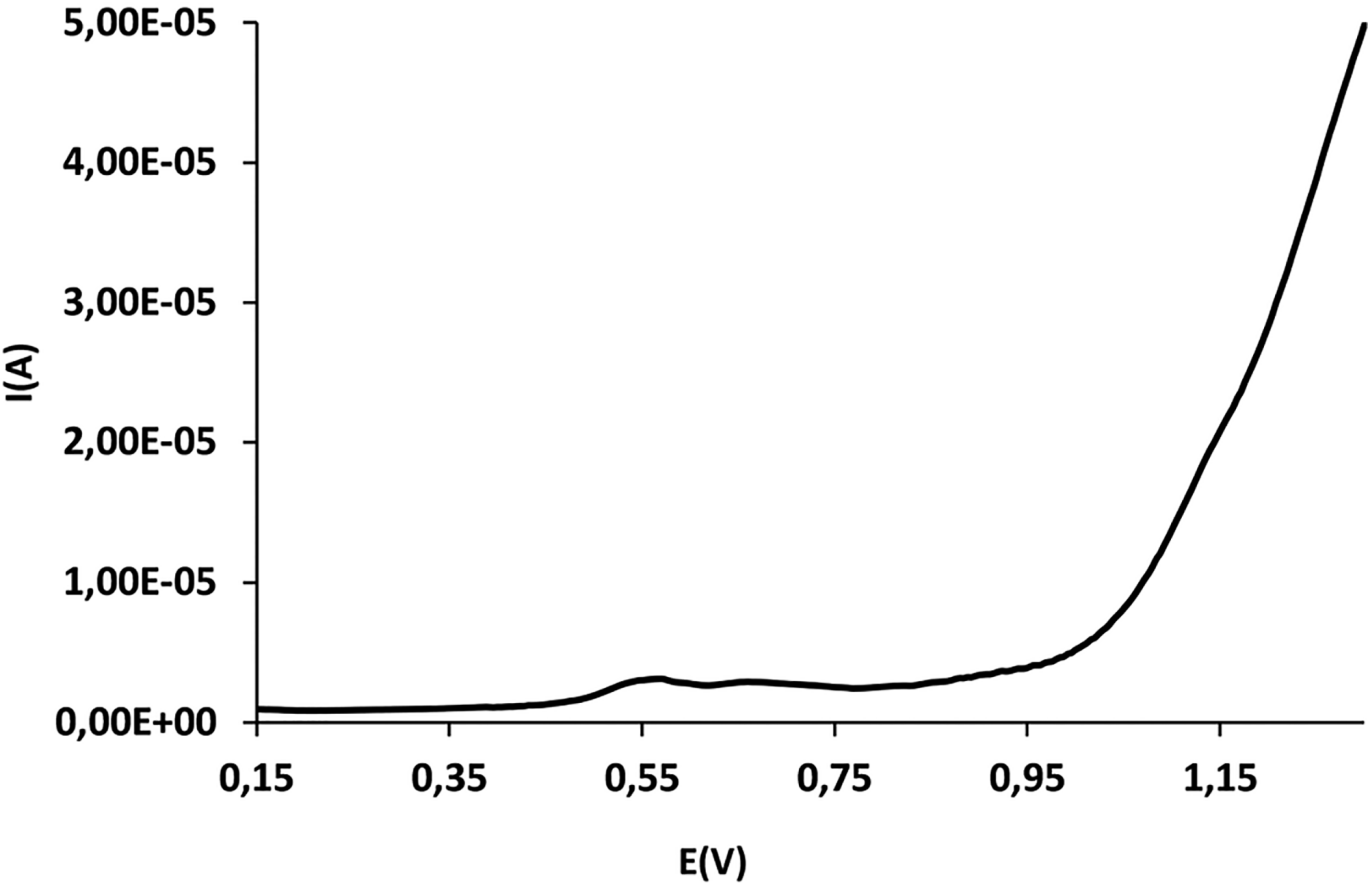
DPV for complex **C4** registered in a phosphate buffer (pH = 7.12) versus Ag/AgCl.


The electrochemical behavior of **C4** was also studied in aqueous solution. The CV curve of **C4** (Figure 3, red line) and DPV curve (Figure 4) exhibit two successive one-electron-oxidation waves at 0.55 and 0.67 V (vs Ag/AgCl), which correspond to Co^III^Ru^III^/Co^III^Ru^II^ and Co^III^Ru^IV^/Co^III^Ru^III^ redox couples, respectively, and are assigned to two proton-coupled electron-transfer (PCET) processes (eqs 1 and 2). These values have been assigned to the Ru^III^/Ru^II^ and Ru^IV^/Ru^III^ couples of the catalytic unit, based in the values presented by the mononuclear [Ru^II^(trpy)(bpy)(H_2_O)]^2+^ compound.^46,47^ The CV curve is followed by another two-electron oxidation wave at 1.19 V (vs Ag/AgCl), which was attributed to the Co^IV^Ru^IV^/Co^III^Ru^IV^ redox couple. In this case, this value is assigned to the Co^IV^/Co^III^ couple of the photoactive unit given the similarity with the potential value of **Na[1]**, whose CV curve presents a Co^IV^/Co^III^ redox wave at *E*_1/2_ = 1.36 V (Figure 3, blue line). For **C4**, this value is 170 mV more cathodic with respect to the potential of **Na[1]**, which indicates a certain and significant electronic coupling between the two units. To check this out, we have measured the redox potentials of **Na[1]** after adding different concentrations of two divalent salts, CaCl_2_ and ZnCl_2_ (Figure S9 and Table S3), to mimic the dipositive charge of the ruthenium complex, and no substantial change in the redox potential of **Na[1]** has been observed. This is consistent with a coupling between the cation and anion in **C4**. Equations 1–4 contain the electrochemical transition reactions corresponding to each redox couple processes at pH = 7.12 with reference electrode Ag/AgCl.

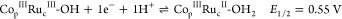
1


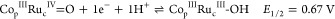
2


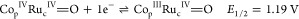
3


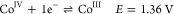
4

Therefore, the observed oxidation potentials indicate that the photogenerated Co^IV^ is able to easily oxidize [Ru^III^-OH]^2+^ to [Ru^IV^=O]^2+^, with a driving force of 520 mV, because the potential for Co^IV^/Co^III^ is high enough compared to the Ru^IV^/Ru^III^ couple. This would be thermodynamically possible in water via PCET processes.^48^ Therefore, on the basis of these studies, it is expected that, for photocatalytic oxidation of organic substrates, **[1]^−^** could act as a good photosensitizer.

Figure 3 shows that there is an ion-pair contact effect, which causes the Co^IV^/Co^III^ redox couple potential to vary near 170 mV under the influence of the ruthenium complex.

### Photocatalytic Oxidation

**C4** is a [Ru][Co]_2_ ion-pair complex in which the photocatalytic system is based on cobalt, an abundant transition metal, whereas the redox part is the ruthenium complex. The photocatalytic oxidation experiments were all carried out by exposing the reaction quartz vials to UV irradiation (2.2 W, λ ∼ 300 nm), for different times, at atmospheric pressure and room temperature. The samples were 5 mL of water, a K_2_CO_3_ solution at pH = 7, and a mixture of **C4** as a substrate, Na_2_S_2_O_8_ as a sacrificial oxidant (air could also do the work),^33^ and 0.005 mol % catalyst. The blank experiments were performed in the absence of catalyst, sacrificial oxidant, or light for 8 h. The results evidenced that a negligible amount of oxidation product was formed, less than 8% in all cases. After irradiation for a specified time (Table 1), the reaction products were extracted with dichloromethane three times, quantified by means of ^1^H NMR spectroscopy, and confirmed by gas chromatography with flame ionization detection (GC-FID) analysis.

**Table 1:**
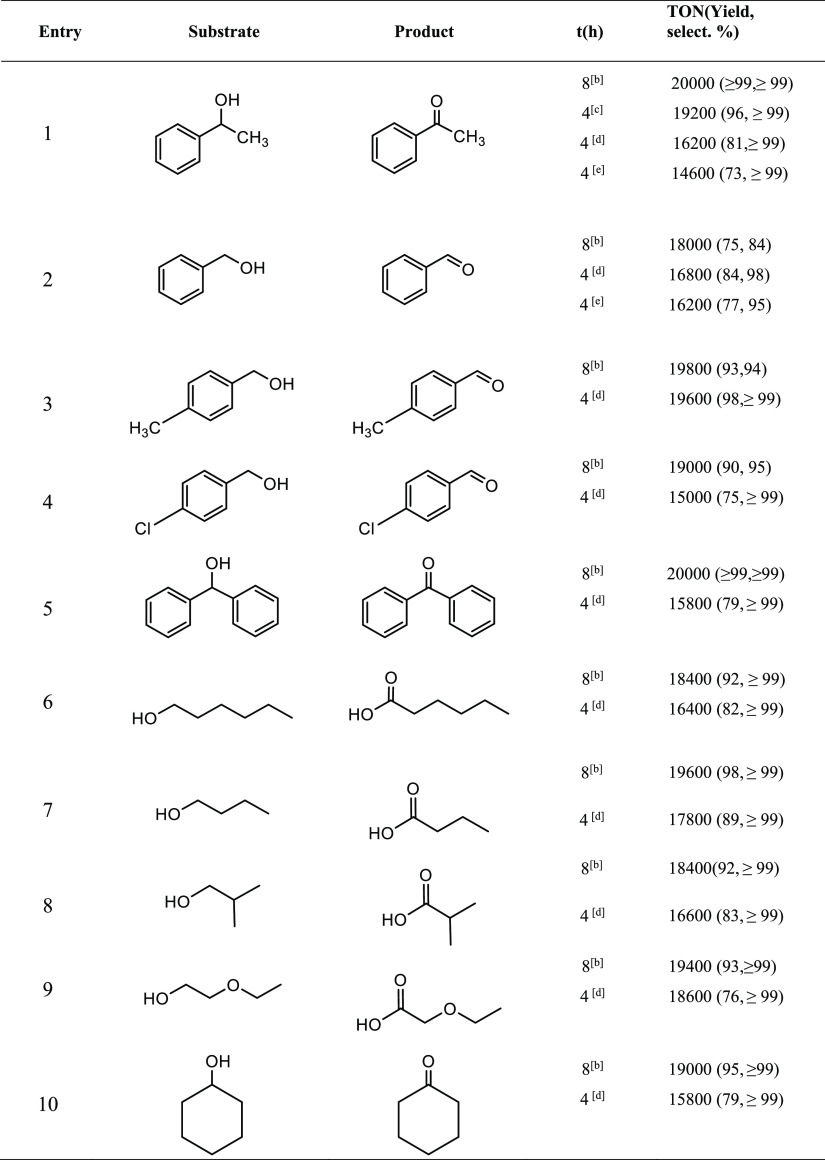
Photooxidation Tests Performed with Ruthenium Cobaltabis(dicarbollide) Complex **C4**^a^

a Conditions: **C4** (0.001 mM), substrate (20 mM), Na_2_S_2_O_8_ (40 mM), 5 mL of a potassium carbonate solution at pH = 7. Ratio 1:20000:40000:

b 8 h of reaction, with previous neutralization after 4 h.

c 4 h of reaction, with previous neutralization after 3 h.

d 4 h of reaction, without neutralization.

e Ratio 1:20000:20000; after 4 h of reaction.

Table 1 displays the catalytic results obtained in the photooxidation of aromatic and aliphatic alcohols, after different times of reaction. In general, high yields of conversion and selectivity have been obtained, even when short reaction times have been used. After 8 h of irraditation and timely neutralization at 4 h, the yields observed in the photooxidation of 1-phenylethanol (entry **1**) and diphenylmethanol (entry **5**), both secondary aromatic alcohols, were remarkably >99%, with selectivity values of >99% and turnover numbers equal to TON = 20000 in both cases. However, the yield values observed for benzyl alcohol are slightly less than the secondary ones. It is worth mentioning that, after 4 h of irradiation, the achieved high selectivity values were to obtain only the corresponding aldehydes. The conversion values enhanced by the presence of electron-donating substituents, such as methyl, on the aromatic ring of the benzyl alcohol (entry **3d**) and decreased with the presence of chloro, an electron-withdrawing substituent (entry **4d**). In the case of both benzyl alcohol and the substituted substrates, the selectivity decreases after 8 h of irradiation, observing alcohol overoxidation toward formation of the corresponding acids. **C4** displays high efficiency by primary and secondary aliphatic alcohols, with yields larger than 76% after 4 h of irradiation and selectivity values of >99%.

1-Hexanol (entry **6**), 1-butanol (entry **7**), isobutyl alcohol (entry **8**) and 2-ethoxyethanol (entry **9**) were converted to the corresponding acids with yields of 82% (4 h) and 92 % (8 h) (entry **6**), 89% (4 h) and 98 % (8 h) (entry **7**), 83% (4 h) and 92 % (8 h) (entry **8**), and 76% (4 h) and 93 % (8 h) (entry **9**), respectively. Conversely, cyclohexanol converted to cyclohexanone in 79% yield after 4 h and 95% after 8 h for **10**. When the amount of oxidizing agent was lowered (ratio applied 1:20000:20000), we observed that the degree of conversion for 1-phenylethanol and benzyl alcohol, after 4 h, is on the same order as the values obtained using a ratio 1:20000:40000 (entries **1e** and **2e**). It should be noticed that the conversion was a little bit less because of the kinetics of the reaction. These results are consistent because 1 mol of S_2_O_8_^2–^ is needed for each 1 mol of alcohol to achieve the oxidizing reaction. We have no evidence that the catalyst has undergone any transformation following catalysis, as has been evidenced in other cases with ruthenium complexes containing trpy.^36^ We performed matrix-assisted laser desorption ionization time-of-flight mass spectrometry (MALDI-TOF MS) of **C4** after the catalytic experiments, and it can be seen that the catalyst remains unchanged after catalysis (Figure S10).

#### Relevance of the History of **C4**

As mentioned in the Introduction, **Na[1]** tends to self-aggregate in water, producing micelles and/or vesicles.^29,30^ Upon the process of dissolving **C4**, the constituent ions would become free; for the vast majority of salts, this is completely independent. However, this allegedly would not be the case here; see also the electrochemical section in which the [Ru−OH_2_]^2+^ affects the redox couple of **[1]^−^**. Persistence even in the water ion-pair coupling between the cation and anion would be possible, which would prevent full dissociation and therefore maintain cooperation. Indeed, no noticeable changes have been observed at the UV–vis spectra in catalytic conditions near 10^–6^ M upon a 10-fold increase in concentration (Figure S11). To evidence the noncovalent bonding relevance between the photosensitizer **[1]^−^** and the redox catalyst, we took advantage of the photooxidation process and studied the yields with the same concentrations of cations and anions but whose origins were different. Thus, we have studied the behavior of a mixture of two separate compounds, [Ru^II^(trpy)(bpy)(H_2_O)](ClO_4_)_2_ (**C2′**) and Na[3,3′-Co(1,2-C_2_B_9_H_11_)_2_] (**Na[1]**), after 4 h of catalysis and using the Co/Ru ratios 1:1 and 2:1, maintaining the same ruthenium concentrations as that for the **C4** photocatalyst. Table 2 displays the results obtained for two substrates, 1-phenylethanol and 4-methylbenzyl alcohol. Table 2 shows that the noncovalent ion pair **C4** displays a better catalytic performance than the equal 1:2 mixture of **C2′** and **Na[1]**, evidencing the advantage of the coupling history between the two compounds, where nonbonding interactions between [Ru^II^(trpy)(bpy)(H_2_O)]^2+^ and **[1]^−^** could facilitate or stimulate ET and, consequently, the efficiency of the cooperative system in the alcohol oxidation. For comparison purposes, the 1:1 ratio of **C2′** and **Na[1]** as well as **C2′** and **Na[1]** alone was also studied. All of these are reported in Table 2, showing a distinct and lower yield than **C4**. Dynamic light scattering (DLS) measurements of catalytic mixtures were done, and the results are displayed in Figure S12. The hydrodynamic radius value of **C4** in the catalytic mixture is *D* = 140.9 nm, which is of the same order as that presented by **Na[1]**,^30^ which indicates the absence of larger aggregates in the medium, despite the presence of the [Ru−OH_2_]^2+^. On the other hand, the value of *D* = 128.4 nm, in the case of using a mixture of a 1:2 ratio of **C2′** and **Na[1]** as catalysts, is less than that for **C4**, indicating to what extent the history of the catalytic medium influences the generated aggregates and hence, although limited, the results.

**Table 2:** Photooxidation Tests Performed with **C4** and **C2′** + **Na[1]**^a^

entry	photocatalyst	substrate	product	TON (yield, selectivity %)
1	**C4**	1-phenylethanol	acetophenone	16200 (81, ≥99)
2	**C2′**	1-phenylethanol	acetophenone	4400 (22, ≥99)
3	**Na[1]**	1-phenylethanol	acetophenone	6200 (62, ≥99)
4	**C2′** + **Na[1]**(1:1)	1-phenylethanol	acetophenone	12800 (64, ≥99)
5	**C2′** + **Na[1]**(1:2)	1-phenylethanol	acetophenone	15200 (76, ≥99)
6	**C4**	4-methylbenzyl alcohol	4-methylbenzaldehyde	19600 (98, ≥99)
7	**C2′**	4-methylbenzyl alcohol	4-methylbenzaldehyde	5200 (25, ≥96)
8	**C2′** + **Na[1]**(1:1)	4-methylbenzyl alcohol	4-methylbenzaldehyde	17200 (70, 82)
9	**C2′** + **Na[1]** (1:2)	4-methylbenzyl alcohol	4-methylbenzaldehyde	18000 (86, 96)

a Conditions: **C4** (0.001 mM); **C2′** and **Na[1]** (0.001 mM) for ratio 1:1 (**C2′**/**Na[1]**); **C2′** (0.001 mM) and **Na[1]** (0.002 mM) for ratio 1:2 (**C2′**/**Na[1]**), substrate (20 mM), Na_2_S_2_O_8_ (40 mM), 5 mL of a potassium carbonate solution at pH = 7. Light irradiation during 4 h.

This study represents the first work, to the best of our knowledge, in which a photoredox catalyst based on an abundant first transition metal (Co^3+^) is noncovalently bonded to an active oxidation catalyst ([Ru^II^(trpy)(bpy)(H_2_O)]^2+^), producing a persistent ion-pair interaction even in water and showing high activity in the photooxidation of alcohols.

**Figure 5:**
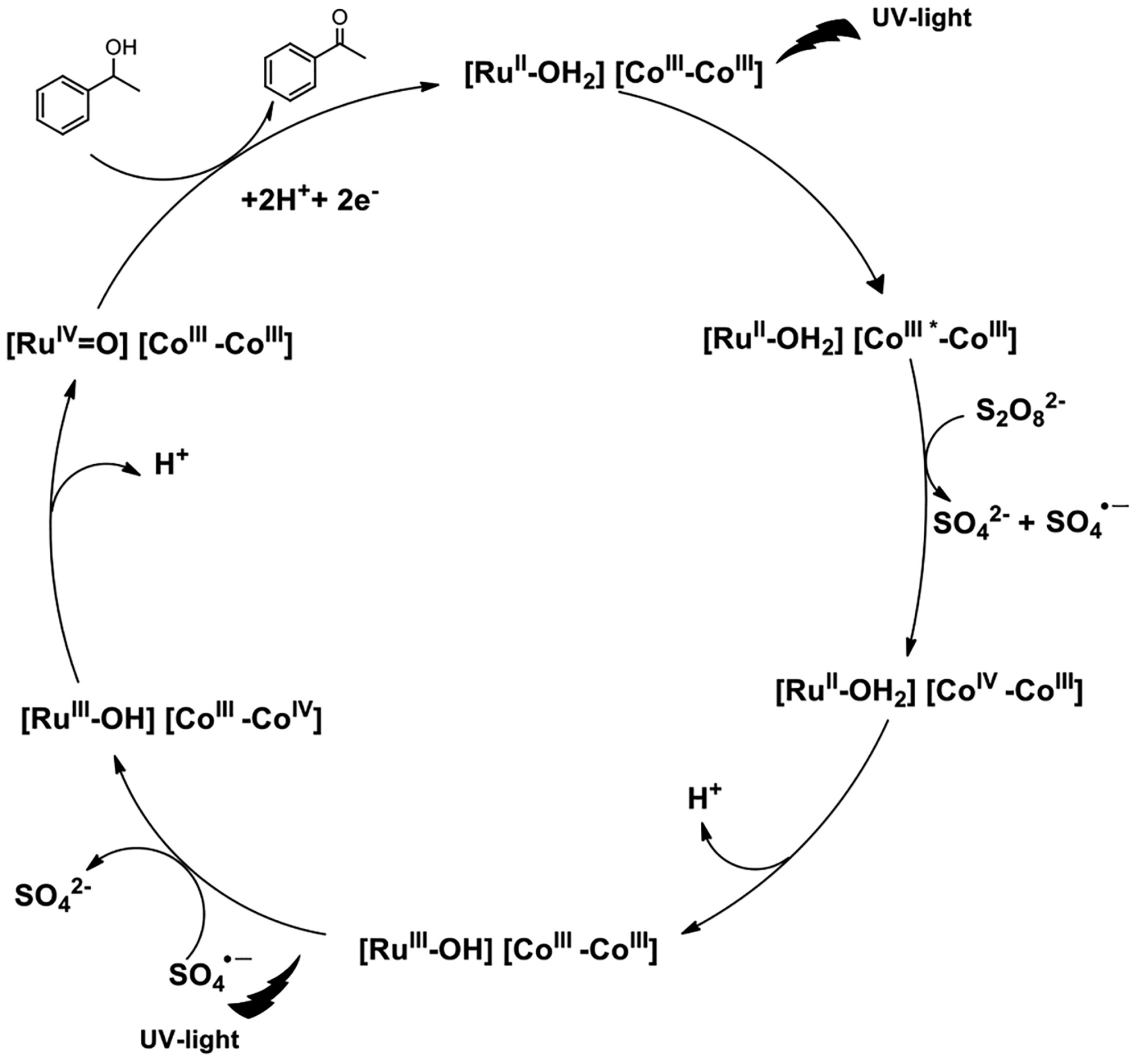
Proposed mechanism for alcohol photooxidation using **C4**.

The observed values of conversion obtained using **C4**, together with the previous electrochemical study, are consistent and support the proposed mechanism shown in Figure 5. Under irradiation, photons are absorbed by the photosensitizer Co_p_^III^ that experiences excitation to form Co_p_^III^*, which undergoes oxidative quenching by the oxidizing agent S_2_O_8_^2–^, generating Co_p_^IV^. This photogenerated strong oxidizing Co^IV^ is able to oxidize Ru_c_^II^-OH_2_ to Ru_c_^III^-OH. Then the SO_4_^•–^ radical oxidizes a new Co_p_^III^ to Co_p_^IV^, which oxidizes Ru_c_^III^-OH to Ru_c_^IV^=O species. The Ru_c_^IV^=O species reacts with the corresponding alcohols to afford the oxidized products, with regeneration of the corresponding catalyst Ru_c_^II^-OH_2_. With the proposed mechanism, the exchange of two electrons and two protons takes place in the photooxidation of alcohols.

## Conclusions

In this work, an air-stable cooperative ion-pair photoredox system, **C4**, formed by cobaltabis(dicarbollide) **[1]^−^** as a light collector, and the aqua ruthenium complex [Ru^II^(trpy)(bpy)(OH_2_)]^2+^ as an ET agent is reported. Both cobaltabis(dicarbollide) and the ruthenium aqua complex are linked by noncovalent interactions, which to a large degree persist even after water dissolution. This represents a step ahead in cooperativity avoiding costly covalent bonding. **C4** displays excellent photoredox catalyst properties in water through PCET. **C4** has been easily made by reaction of the chlorido ruthenium (II) complex with **Ag[1]** in water/acetone (1:1) under reflux. The recrystallization of the aqua complex **C4** in acetonitrile yielded **C5** in which the aqua ligand was substituted by CH_3_CN, as shown in X-ray diffraction. The electrochemical studies evidence a significant electronic coupling between the two units in the noncovalent ion-pair ruthenium cobaltabis(dicarbollide) compound and that the photogenerated Co^IV^ can easily oxidize [Ru^III^-OH]^2+^ to [Ru^IV^=O]^2+^ in water via PCET processes; therefore, the metallacarborane can act as a good photosensitizer for the photocatalytic oxidation of organic substrates in this cooperative system.

We have highlighted the capacity of **C4** to perform as an excellent cooperative photoredox catalyst in the oxidation of alcohols in water, using a catalyst load of 0.005 mol %, achieving high yields even when short reaction times of irradiation have been used. In some cases, a high turnover number (TON = 20000) has been observed.

The Co^IV/III^ redox couple of **[1]^−^** in water differed by 170 mV when **[1]^−^** had Na^+^ as the cation versus when [Ru−OH_2_]^2+^ was the cation. In solution, having one or the other cation should not influence the potential, unless there were bonds that lasted even after dissolution. This led us to study two identical solutions in **[1]^−^** and **[Ru]**: one resulting from dissolving **C4** and the other from dissolving the precursors in water. It was remarkable to find that **C4** displays a better catalytic performance than the mixtures of **C2′** and **Na[1]**, evidencing that the noncovalent bonding existing in **C4** facilitates or stimulates ET between the photosensitizer and catalyst and, consequently, efficiency of the cooperative system in the photooxidation process. To the best of our knowledge, cooperative systems with this type of interaction have not been described and represent a step forward in getting cooperativity avoiding covalent bonding. In most of the systems displayed in the literature, both the photoredox catalyst and the catalyst are connected through an organic linker or the metals of both share the same ligand. A possible pathway has been proposed.

## Experimental Section

### Materials, Instrumentation, and Measurements

Na[Co(1,2-C_2_B_9_H_11_)_2_] (**Na[1]**),^31^ and Ag[Co(1,2-C_2_B_9_H_11_)_2_] (**Ag[1]**, **C3**)^38^ were synthesized from commercial Cs[Co(1,2-C_2_B_9_H_11_)_2_] (**Cs[1]**; Katchem Spol.sr.o), following the methods described in the literature.^38^ [Ru^III^Cl_3_(trpy)] (**C1**),^49^ [Ru^II^Cl(trpy)(bpy)]Cl (**C2**), and [Ru^II^ (trpy)(bpy)(OH) _2_](ClO_4_)_2_ (**C2′**)^46,47^ were also prepared according to literature procedures. All synthetic manipulations were performed under a nitrogen atmosphere using vacuum-line techniques.

All reagents used in the present work were obtained from Aldrich Chemical Co. and used without further purification. Reagent-grade organic solvents were obtained from SDS, and high-purity deionized water was obtained by passing distilled water through a Nanopure Milli-Q water purification system.

UV–vis spectroscopy was performed on a Cary 50 Scan (Varian) UV–vis spectrophotometer with 1 cm quartz cells or with an immersion probe of 5 mm path length. NMR spectra have been recorded with a Bruker ARX 300 instrument, and ^1^H NMR spectra were recorded in acetone-*d*_6_. Chemical shift values were referenced to SiMe_4_. Elemental analyses were performed using a CHNS-O EA-1108 elemental analyzer from Fisons. Electrospray ionization mass spectrometry (ESI-MS) experiments were performed on a Navigator liquid chromatography (LC)/MS chromatograph from Thermo Quest Finnigan, using acetonitrile as the mobile phase. CV or DPV was performed on an IJ-Cambria 660C potentiostat using a three-electrode cell. A glassy carbon electrode (3 mm diameter) from BAS was used as the working electrode and Ag/AgCl as the reference electrode. All cyclic voltammograms presented in this work were recorded under a nitrogen atmosphere. The complexes were dissolved in deoxygenated solvents containing the necessary amount of [*n*-Bu_4_N][PF]_6_ (TBAH) as the supporting electrolyte to yield a 0.1 M ionic strength solution. All *E*_1/2_ values reported in this work were estimated from CV experiments as an average of the oxidative and reductive peak potentials [(*E*_pa_ + *E*_pc_)/2]. Unless explicitly mentioned, the concentration of the complexes was approximately 1 mM.

GC was performed with a GC-2010 gas chromatograph from Shimadzu, equipped with an Astec CHIRALDEX G-TA column [30 m × 0.25 mm (i.d.); FID detector, 250 °C; injection, 250 °C; carrier gas, helium; rate, 1.57 mL min^–1^; area normalization]. Product analyses in the catalytic experiments were performed by GC with biphenyl as the internal standard.

#### Crystallographic Data Collection and Structure Determination of **C5**

Measurement of the **C5** crystals was performed on a Bruker Smart Apex CCD diffractometer using graphite-monochromated Mo Kα radiation (λ = 0.71073 Å) from an X-ray tube: data collection, *SMART*, version 5.631 (Bruker AXS 1997–2002); data reduction, *SAINT+*, version 6.36A (Bruker AXS 2001); absorption correction, *SADABS*, version 2.10 (Bruker AXS 2001); structure solution, SHELXTL, version 6.14 (Bruker 2003); structure refinement, *SHELXL-2018/3* (Sheldrick, 2018). The crystallographic data as well as details of the structure solution and refinement procedures are reported in the Supporting Information. CCDC 2058809 for **C5** contains the supplementary crystallographic data for this paper.

##### Synthesis of [Ru^II^(trpy)(bpy)(H_2_O)][3,3′-Co(1,2-C_2_B_9_H_11_)_2_]_2_ (**C4**)

A 90 mg sample of **C2** and a 173 mg sample of **Ag[1]** were dissolved in 60 mL of acetone.water (1:1), and the resulting solution was refluxed for 3 h. Then, AgCl was filtered off through a frit containing Celite. The volume of the solution was reduced, and the mixture was chilled in a refrigerator for 48 h. The dark orange precipitate was collected on a frit, washed with cold water and anhydrous ethyl ether, and then vacuum-dried. Yield: 134.69 mg (89.93%). Anal. Found (calcd) for C_33_ H_65_B_36_N_5_Co_2_ORu·1.5H_2_O·2Et_2_O: C, 37.04 (36.99); H, 6.24 (6.66); N, 5.19 (5.26). ^1^H NMR (acetone-*d*_6_, 400 MHz): δ 9.87 (d, 1H, ^3^*J*_H–H_ = 5.6 Hz, 1H, H1), 8.98 (d, 1H, ^3^*J*_H–H_ = 8.1 Hz, 1H, H7), 8.91 (d, ^3^*J*_H–H_ = 8.0 Hz, 2H, H17, H19), 8.76 (d, ^3^*J*_H–H_ = 8.1 Hz, 2H, H14, H22), 8.64 (d, ^3^*J*_H–H_ = 8.1 Hz, 1H, H4), 8.52 (t, ^3^*J*_H–H_ = 8.1 Hz, 1H, H8), 8.43 (t, ^3^*J*_H–H_ = 8.1 Hz, 1H, H18), 8.23 (t, ^3^*J*_H–H_ = 7.1 Hz, 1H, H9), 8.16 (t, ^3^*J*_H–H_ = 8.1 Hz, 2H, H13, H23), 8.04(d, ^3^*J*_H–H_ = 5.6 Hz, 2H, H11, H25), 7.88 (t, ^3^*J*_H–H_ = 8.4 Hz, 1H, H3), 7.55 (ddd, ^3^*J*_H–H_ = 7.9 Hz, 8.0 Hz, ^4^*J*_H–H_ = 1.3 Hz, 3H, H10, H12, H24), 7.17 (t, ^3^*J*_H–H_ = 7.0 Hz, 1H, H2), 5.83 (s, 2H, Ru–OH_2_), 3.98 (s, 8H, C_c_–H). ^1^H{^11^B} NMR (acetone-*d*_6_, 400 MHz): δ 3.98 (s, 8H, C_c_–H), 3.41 (s, 4B–H, B8, B8′), 3.16 (s, 4B–H, B10, B10′), 2.75(s, 8B–H, B4, B4′, B7, B7′), 1.96 (s, 8B–H, B9, B9′, B12, B12′), 1.68 (s, 4B–H, B6, B6′), 1.59 (s, 8B–H, B5, B5′, B11, B11′). ^11^B NMR (acetone-*d*_6_, 128 MHz): δ 6.31 (d, 4B, *J*_B–H_ = 144.3 Hz, B–H), 1.13 (d, 4B, *J*_B–H_ = 143.5 Hz, B–H), −5.87 (m, 16B, B–H), −17.45 (d, 8B, *J*_B–H_ = 156.0 Hz, B–H), −22.90 (d, 4B, *J*_B–H_ = 165.3 Hz, B–H). ^11^B{^1^H} NMR (acetone-*d*_6_, 128 MHz): δ 6.33 (s, 4B, B8, B8′), 1.19 (s, 4B, B10, B10′), −5.87 (d, *J*_B–B_ = 95.4 Hz 16B, B4, B7, B4′, B7′, B9, B12, B9′, B12′), −17.40 (s, 8B, B5′, B11′, B5, B11), −22.84 (s, 4B, B6′, B6). ^13^C{^1^H} NMR (acetone-*d*_6_, 100 MHz): δ 159.42, 158.98, 153.69, 153.55, 150.66, 138.74, 138.46, 128.32, 127.91, 126.68, 126.50, 124.27, 124.17, 123.63, 123.47 (C Ru trpy-bpy), 51.11 and 50.89 (C_c_). IR (ν, cm^–1^): 3039, 2922, 2854, 2531, 1601, 1446, 1464,1384, 1095, 1016, 980, 884, 761. *E*_1/2_ (CH_2_Cl_2_ + 0.1 M TBAH): Co^III/II^, −1.37 V; Co^IV/III^, 1.38 V; Ru^III/II^, 0.70 V; Ru^IV/III^, 1.06 V (vs Ag/AgCl). UV–vis [CH_2_Cl_2_, 1.16 × 10^–5^ M; λ_max_, nm (ε, M^–1^ cm^–1^)]: 279 (37297), 292 (42727), 325 (66493), 392 (9686), 477 (8362). ESI-MS: *m*/*z* 814.4 (100%, [M – cosane – H_2_O]^+^), 831.4 (31%, [M – cosane]^+^).

##### Synthesis of [Ru^II^(trpy)(bpy)(CH_3_CN)][3,3′-Co(1,2-C_2_B_9_H_11_)_2_]_2_ (**C5**)

By recrystallization of **C4** in an acetonitrile solution, yellow needles suitable for X-ray diffraction were obtained corresponding to complex **C5**. UV–vis [CH_3_CN, 1.16 × 10^–5^ M; λ_max_, nm (ε, M^–1^ cm^–1^)]: 287 (59852), 308 (31314), 333 (11997), 464 (5774).

### Photocatalytic Studies

A quartz tube containing an aqueous solution (5 mL) at pH = 7 (K_2_CO_3_) with **C4** or **C2′** as the catalyst, alcohol as the substrate, and Na_2_S_2_O_8_ as the sacrificial acceptor was exposed to UV light (2.2 W, λ = 300 nm) for different times. The complex/substrate/sacrificial oxidant ratios used (1:20000:40000 and 1:20000:20000 corresponding to concentrations of 0.001:20:40 mM and 0.001:20:20 mM) were varied according to the study. For each experiment, a light reactor supplied light illumination with 12 lamps that produce UVA light at room temperature. The resulting solutions were extracted with CH_2_Cl_2_ three times. The solution was dried with anhydrous sodium sulfate, and the solvent was evaporated under reduced pressure. To check the reproducibility of the reactions, all of the experiments were carried out in triplicate. The reaction products were quantified and characterized by ^1^H NMR spectroscopy using tetramethylsilane as the internal standard in the case of primary aromatic alcohols and confirmed by gas chromatography.

## Supporting Information Available

Supplementary crystallographic information, spectroscopic characterization (IR, NMR, UV–vis, and ESI-MS spectra) and additional electrochemical characterization, MALDI-TOF-MS of **C4** after photooxidation, and UV–vis and DLS of catalytic mixtures (PDF)

## Accession Codes

CCDC 2058809 contains the supplementary crystallographic data for this paper. These data can be obtained free of charge via www.ccdc.cam.ac.uk/data_request/cif, or by emailing data_request@ccdc.cam.ac.uk, or by contacting The Cambridge Crystallographic Data Centre, 12 Union Road, Cambridge CB2 1EZ, UK; fax: +44 1223 336033.
