# Supplementary material for: Aqueous Persistent Noncovalent Ion-Pair Cooperative Coupling in a Ruthenium Cobaltabis(dicarbollide) System as a Highly Efficient Photoredox Oxidation Catalyst
Source: Inorg Chem. 2021 Jun 7;60(12):8898–907. doi: 10.1021/acs.inorgchem.1c00751 (PMC8485323; doi:10.1021/acs.inorgchem.1c00751)

SUPPORTING INFORMATION

Aqueous Persistent Noncovalent Ion-Pair Cooperative Coupling in a Ruthenium Cobaltabis(dicarbollide) System as a Highly Efficient Photoredox Oxidation Catalyst

Isabel Guerrero,[a,b] Clara Viñas,[a] Xavier Fontrodona,[b] Isabel Romero,[b]* Francesc Teixidor[a]*

[a]Institut de Ciencia de Materials de Barcelona, Consejo superior de Investigaciones Científicas, E-08193 Bellaterra, Spain.

[b]Departament de Química and Serveis Tecnics de Recerca, Universitat de Girona, E-17003 Girona, Spain.

*Correspondence to: teixidor@icmab.es; marisa.romero@udg.edu

TABLE OF CONTENTS

Table S1. Crystal Data for X-ray structure of complex **C5**.

Table S2. Selected bond lengths (Å) and angles (°) for **C5** complex.

Table S3. Potential values of complex **Na[1]**, corresponding to the couple Co^{IV}/Co^{III} after the addition of different concentrations of a) $CaCl_2$ and b) $ZnCl_2$.

Figure S1. a) Dihydrogen bonds C-H···H-B interactions between cationic and anionic moieties in **C5**; b) packing displayed by **C5.**

Figure S2. IR spectrum of **C4.**

Figure S3. a) $^1H\{^{11}B\}$-NMR and b) $^{11}B\{^1H\}$-NMR spectra of Ag[1] **C3** compound.

Figure S4. a) ^1H-NMR; b) $^1H\{^{11}B\}$-NMR and magnification of cobaltabisdicarbollide anion in **C4**; c) $^{11}B\{^1H\}$-NMR and d) COSY NMR spectra of **C4** compound in acetone-d_6.

Figure S5. UV-visible of a) Ag[1], **C3** and b) **C2'** (black line) and **C4** (dash dotted line) in phosphate buffer at 7.02 pH.

Figure S6. **Figure S6.** CV of a) Ag[1] **C3** in CH_3CN + 0.1 M TBAH vs Ag; and b) **C4** in CH_2Cl_2 + 0.1 M TBAH vs Ag/AgCl.

Figure S7. CV for complex **Na[1]** registered in a phosphate buffer (pH= 7.12) vs Ag/AgCl.

Figure S8. CV for complex **C4** registered in a phosphate buffer (pH= 7.12) vs Ag/AgCl.

Figure S9. CV of complex **Na[1]** (1 mM) registered in a phosphate buffer (pH= 7.12) vs Ag/AgCl, before and after the addition of 1 mM of a) $CaCl_2$ and b) $ZnCl_2$. The blue and purple inserts show dI/dE to better appreciate the position of the couple Co^{IV}/Co^{III}.

Figure S10. MALDI of **C4** after the photooxidation of 1-phenylethanol. $[3,3'\text{-}Co(1,2\text{-}C_2B_9H_{11})_2]^-$ =324.1 (m/z) and $[3,3'Co(1,2\text{-}C_2B_9H_{11})(1,2\text{-}C_2B_9H_{11}O)]^-$ =340.1 (m/z).

Figure S11. UV-Visible spectra of mixtures of **C2':Na[1]** (1:1) (black line), (1:2) (dotted line) and **C4** (dash dotted line) at a)3×10^{-6} and b) 0.33×10^{-6} M.

Figure S12. DLS of catalytic mixtures using a) **C4** as catalyst and b) **C2'** and **Na[1]** (1:2) as catalyst.

Figure S13. ESI-MS spectra of **C4**.

Table S1. Crystal Data for X-ray structure of complex **C5**.

	**[C5]**
Empirical formula	$C_{35}H_{66}N_6B_{36}Co_2Ru$
Formula weight	1179.02
Crystal system	Triclinic
Space group	P-1
a [Å]	14.2721(17)
b [Å]	14.9427(17)
c [Å]	15.5782(19)
α [º]	114.464(2)
β [º]	106.404(2)
γ [º]	99.788(2)
V [$Å^3$]	2739.8(6)
Formula Units/Cell	2
$\rho_{calc.}$ [g cm^{-3}]	1.429
μ [mm^{-1}]	0.910
*R*1[a], [I > 2σ(I)]	0.0716
wR_2[b] [all data]	0.1639

[a] $R_1 = \Sigma||F_o| - |F_c||/\Sigma|F_o|$

[b] $wR_2 = [\Sigma\{w(F_o^2-F_c^2)^2\}/\Sigma\{w(F_o^2)^2\}]^{½}$,where $w = 1/[\sigma^2(Fo^2) + (0.0042P)^2]$ and $P=(F_o^2+2F_c^2)/3$

Table S2. Selected bond lengths (Å) and angles (°) for **C5** complex.

Ru(1)-N(1)	2.034(6)	Co(3C)-C(2C)	2.049(7)	N(1)-Ru(1)-N(2)	78.9(2)
Ru(1)-N(2)	2.069(6)	Co(3C)-B(4C)	2.066(7)	N(1)-Ru(1)-N(3)	92.6(2)
Ru(1)-N(3)	2.067(5)	Co(3C)-C(1C)	2.022(7)	N(1)-Ru(1)-N(4)	92.3(2)
Ru(1)-N(4)	1.961(5)	Co(3C)-C(2C)_a	2.049(7)	N(1)-Ru(1)-N(5)	90.1 (2)
Ru(1)-N(5)	2.079(5)	Co(3C)-C(1C)_a	2.022(7)	N(1)-Ru(1)-N(6)	174.5(2)
Ru(1)-N(6)	2.035(6)	Co(3D)-B(4D)_b	2.070(8)	N(2)-Ru(1)-N(3)	97.4(2)
Co(3A)-C(2A)	2.046(7)	Co(3D)-B(7D)_b	2.077(8)	N(2)-Ru(1)-N(4)	170.7 (2)
Co(3A)-C(1A)	2.065(7)	Co(3D)-B(7D)	2.077(8)	N(2)-Ru(1)-N(5)	103.6(2)
Co(3A)-B(7A)	2.062(8)	Co(3D)-C(2D)_b	2.041(7)	N(2)-Ru(1)-N(6)	96.1(2)
Co(3A)-B(4B)	2.093(9)	Co(3D)-C(2D)	2.041(7)	N(3)-Ru(1)-N(4)	79.9(2)
Co(3A)-B(7B)	2.080(7)	Co(3D)-B(4D)	2.070(8)	N(3)-Ru(1)-N(5)	158.9(2)
Co(3A)-B(8A)	2.097(9)	Co(3D)-C(1D)	2.041(8)	N(3)-Ru(1)-N(6)	90.3(2)
Co(3A)-C(1B)	2.045(8)	Co(3D)-C(1D)_b	2.041(8)	N(4)-Ru(1)-N(5)	79.1(2)
Co(3A)-C(2B)	2.040(7)	Co(3D)-B(8D)_b	2.103(8)	N(4)-Ru(1)-N(6)	92.8(2)
Co(3A)-B(4A)	2.098(9)	Co(3D)-B(8D)	2.103(8)	N(5)-Ru(1)-N(6)	88.9(2)
Co(3C)-B(4C)_a	2.066(7)				

Table S3. Potential values of complex **Na[1]**, corresponding to the couple Co^{IV}/Co^{III} after the addition of different concentrations of a) $CaCl_2$ and b) $ZnCl_2$.

a)

Entry	[Na[1]](M)	$E^{CoIV/III}$(Na[1]) (V)[a]	$[CaCl_2]_{added}$(M)	$E^{CoIV/III}$(Na[1]+$CaCl_2$)[a](V)
**1**	$1x10^{-3}$	1.36	$2.5x10^{-4}$	1.354
**2**	$1x10^{-3}$	1.36	$5x10^{-4}$	1.346
**3**	$1x10^{-3}$	1.36	$1x10^{-3}$	1.352

a. potentials registered in a phosphate buffer (pH=7.12) vs Ag/AgCl.

b)

Entry	[Na[1]](M)	$E^{CoIV/III}$(Na[1]) (V)[a]	$[ZnCl_2]_{added}$(M)	$E^{CoIV/III}$ (Na[1]+$ZnCl_2$)[a](V)
**1**	$1x10^{-3}$	1.36	$2.5x10^{-4}$	1.348
**2**	$1x10^{-3}$	1.36	$5x10^{-4}$	1.368
**3**	$1x10^{-3}$	1.36	$1x10^{-3}$	1.338

a. potentials registered in a phosphate buffer (pH=7.12) vs Ag/AgCl.

Figure S1. a) Dihydrogen bonds C-H···H-B interactions between cationic and anionic moieties in **C5**; b) packing displayed by **C5.**

a)

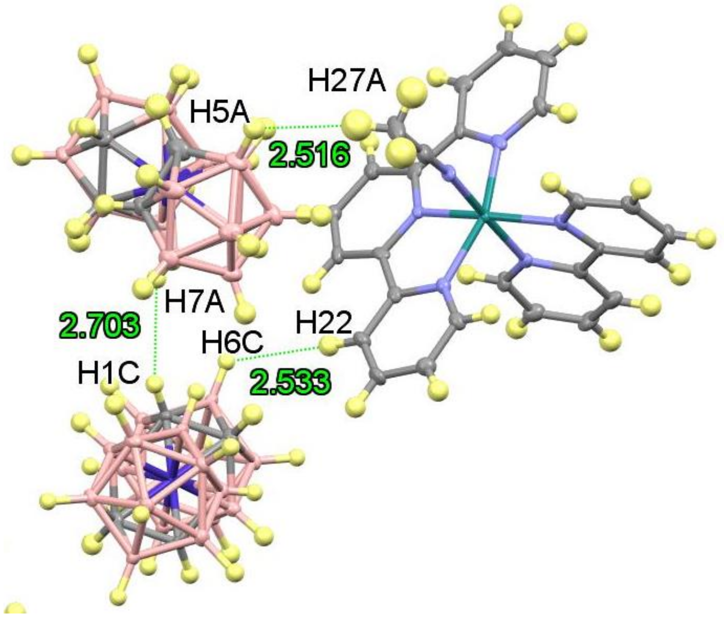

b)

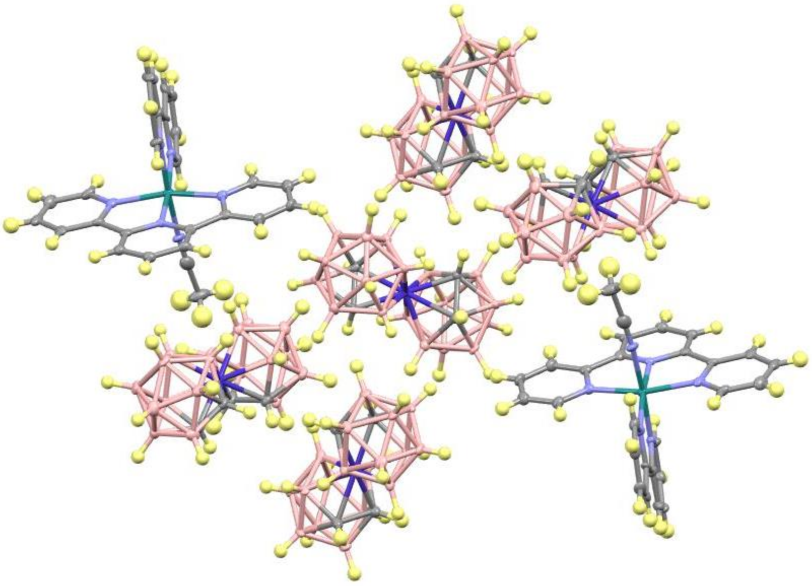

Figure S2. IR spectrum of **C4.**

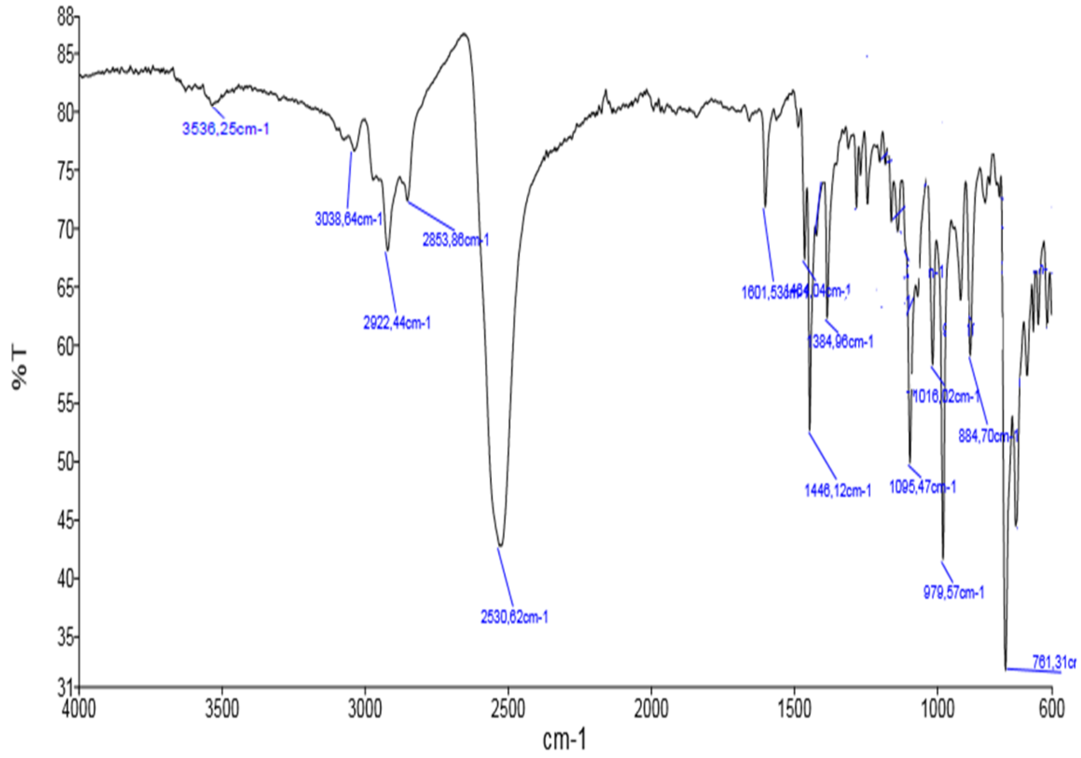

Figure S3. a) $^{1}H\{^{11}B\}$-NMR and b) $^{11}B\{^{1}H\}$-NMR spectra of Ag[1] **C3** compound in acetone-d_6.

a)

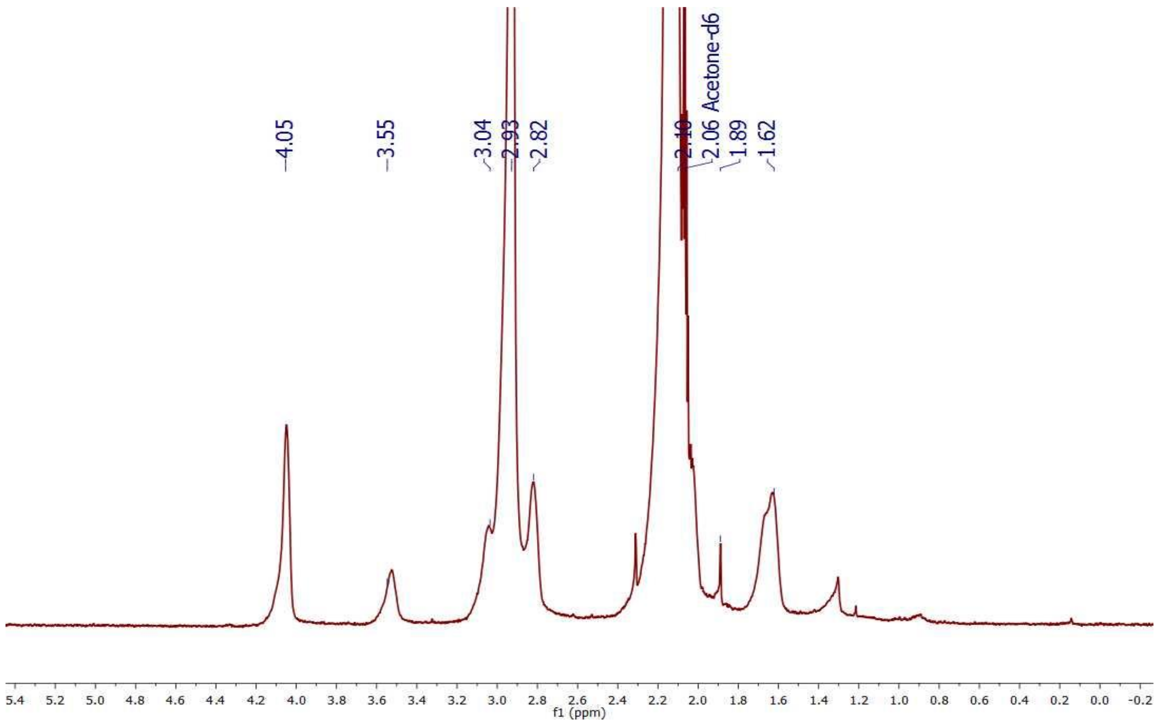

b)

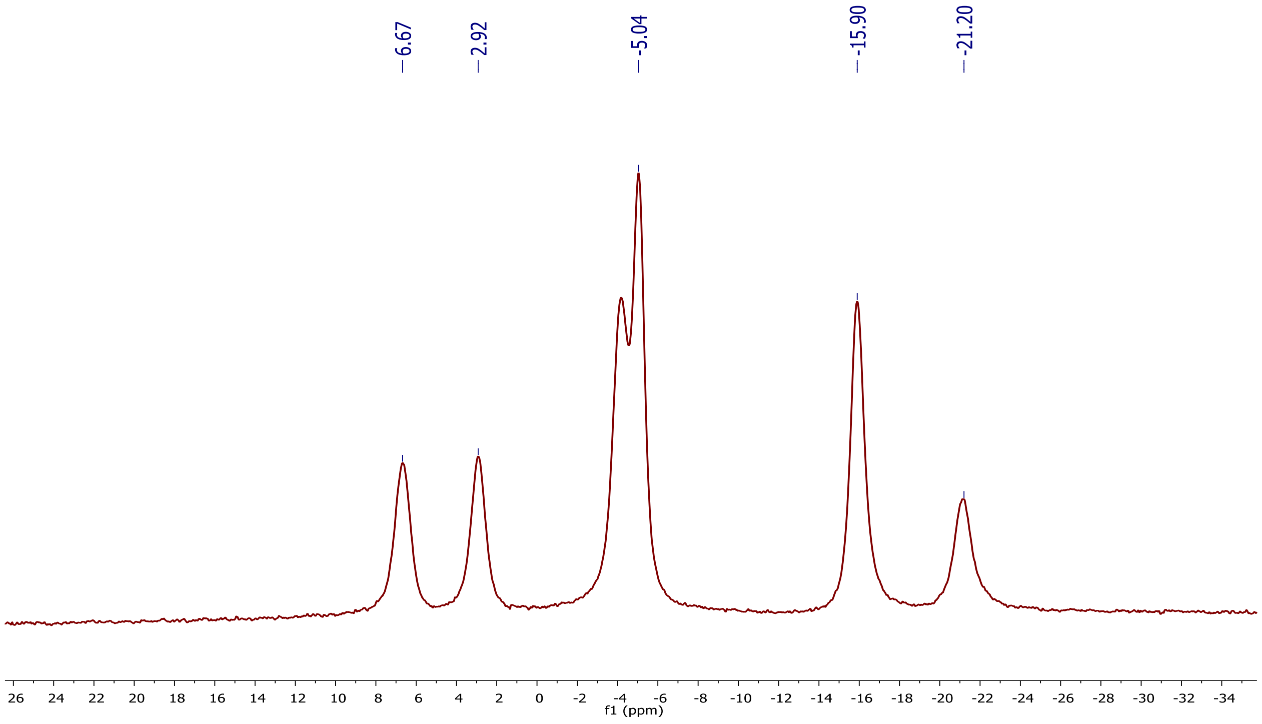

Figure S4. a) ^{1}H-NMR; b) ^{1}H{^{11}B}-NMR; c) ^{11}B{^{1}H}-NMR and d) COSY NMR spectra of cobaltabisdicarbollide **C4** compound in acetone-d$_6$.

a)

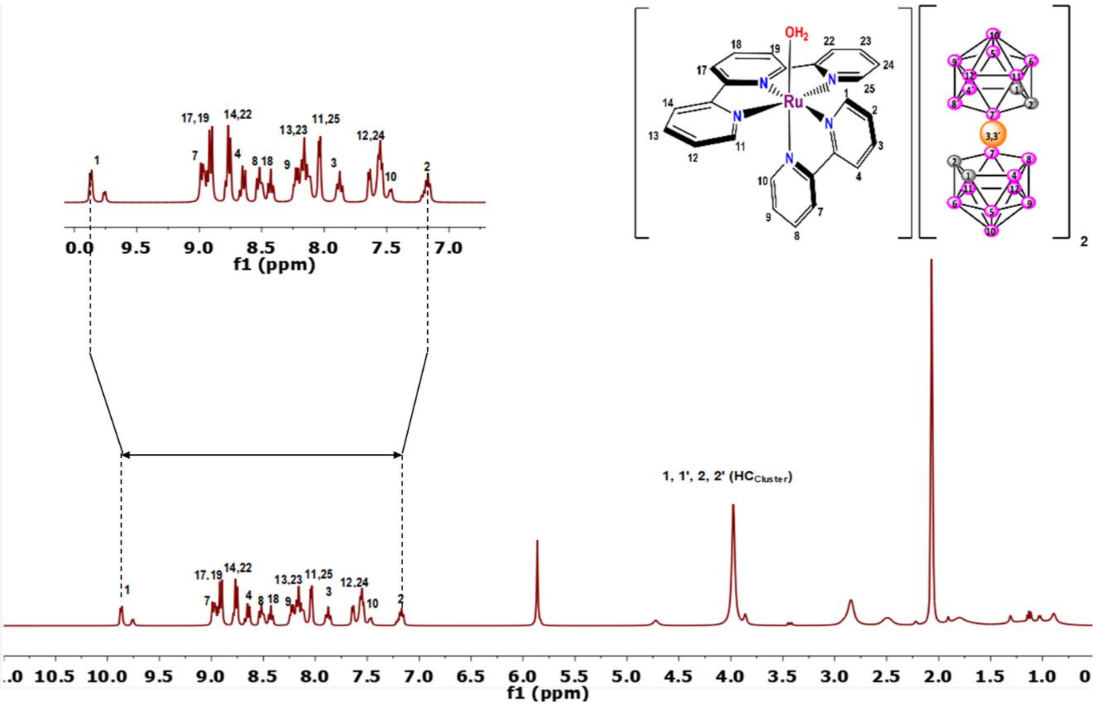

b)

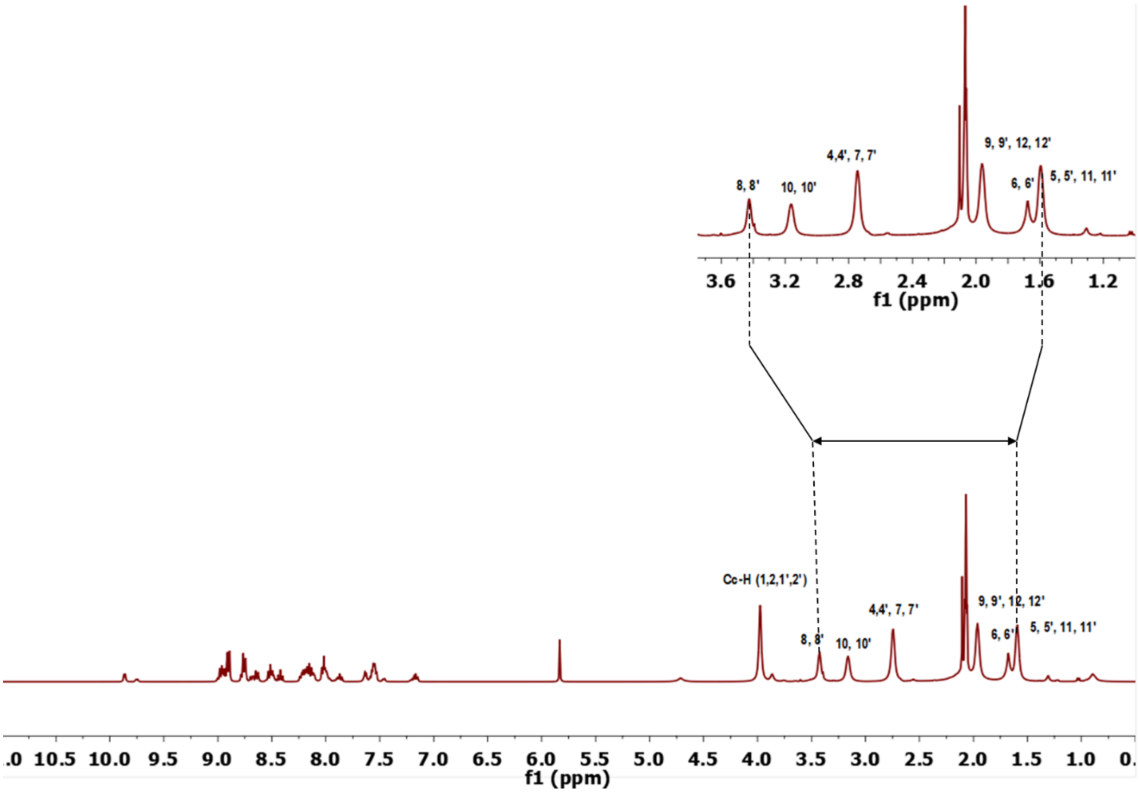

c)

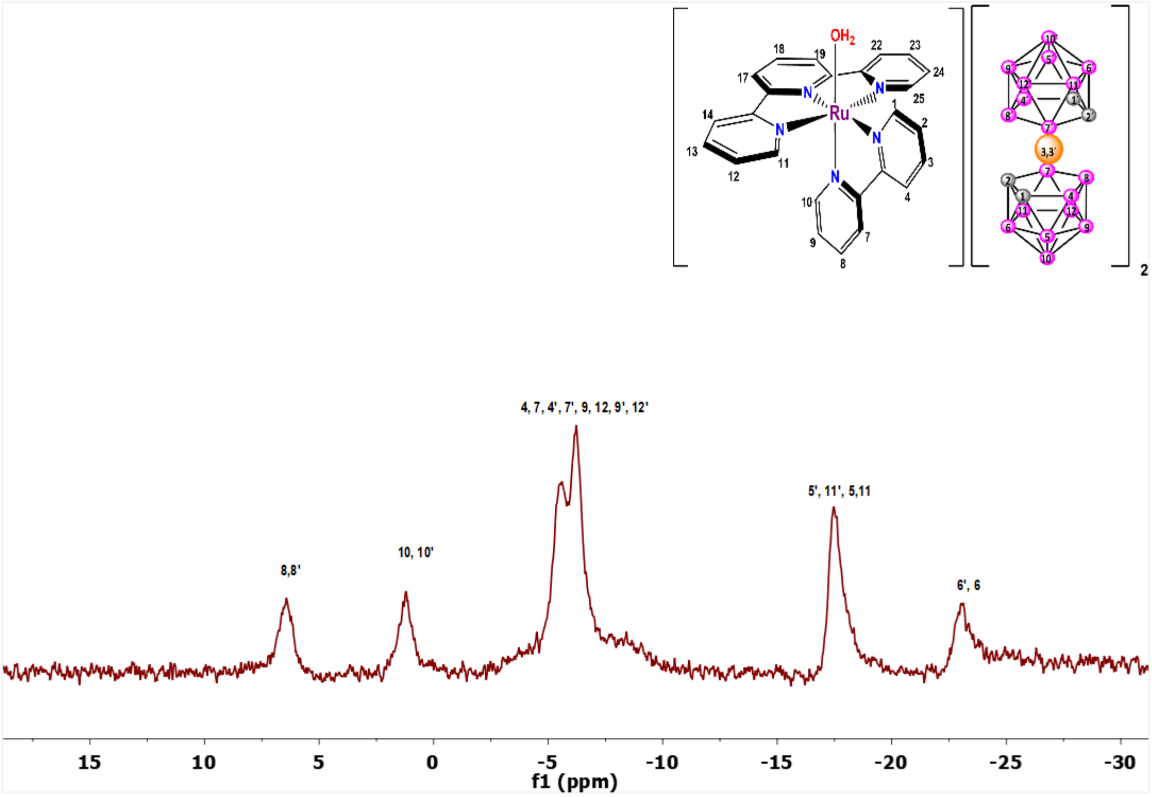
OH2
Ru
8,8'
10, 10'
4, 7, 4', 7', 9, 12, 9', 12'
5', 11', 5,11
6', 6
15
10
5
0
-5
-10
-15
-20
-25
-30
f1 (ppm)

d)

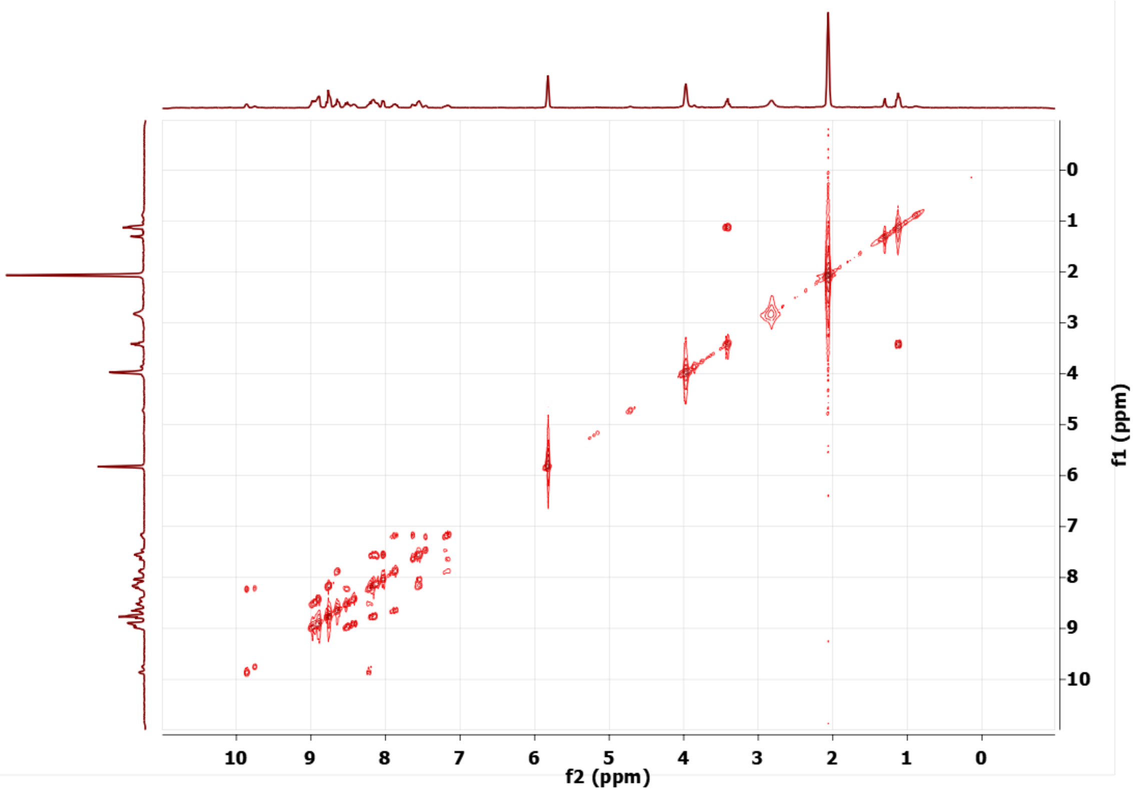
f2 (ppm)
f1 (ppm)

Figure S5. UV-visible of a) Ag[1], **C3** and b) **C2'** (black line) and **C4** (dash dotted line) in phosphate buffer at 7.02 pH.

a)

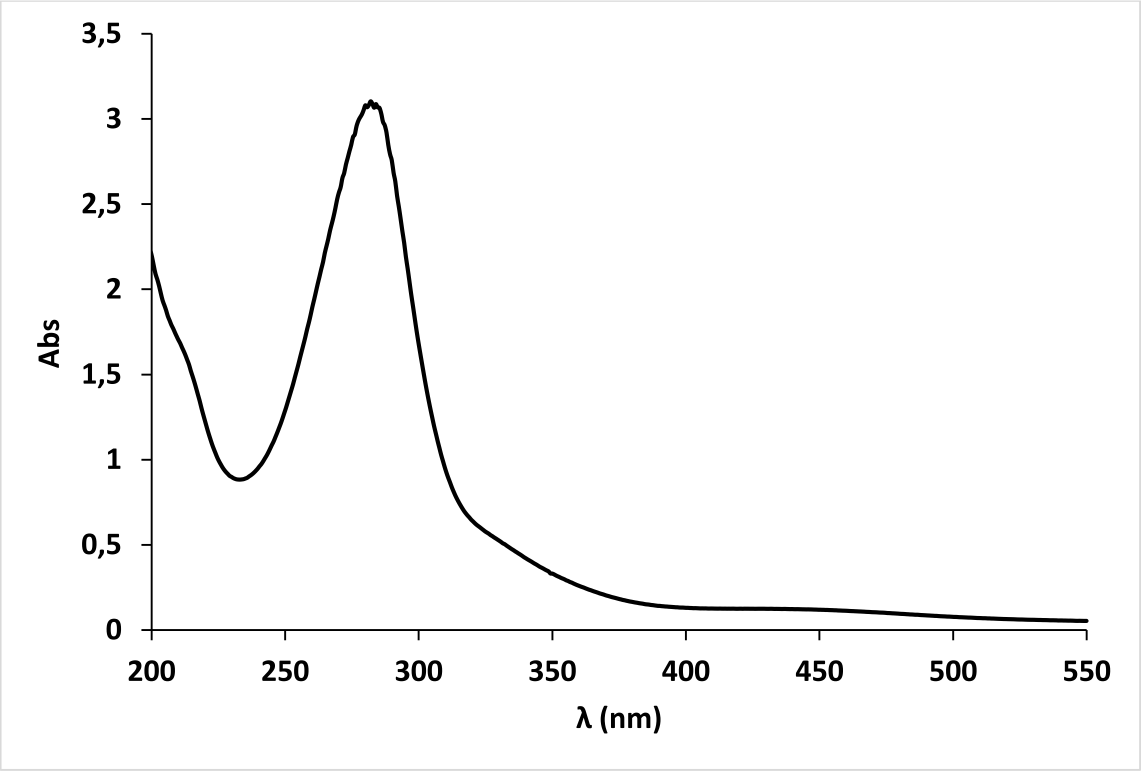

b)

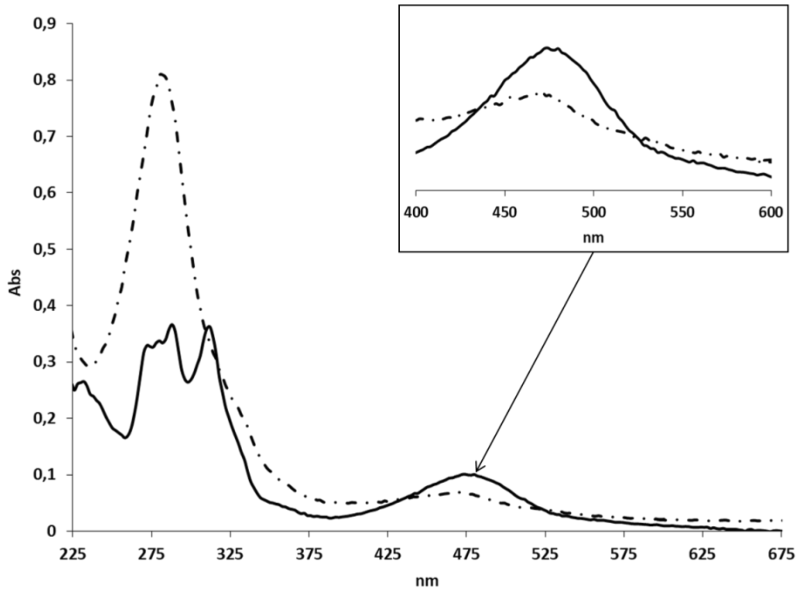

Figure S6. CV of a) Ag[1] **C3** in CH_3CN + 0.1 M TBAH vs Ag; and b) **C4** in CH_2Cl_2 + 0.1 M TBAH vs Ag/AgCl.

a)

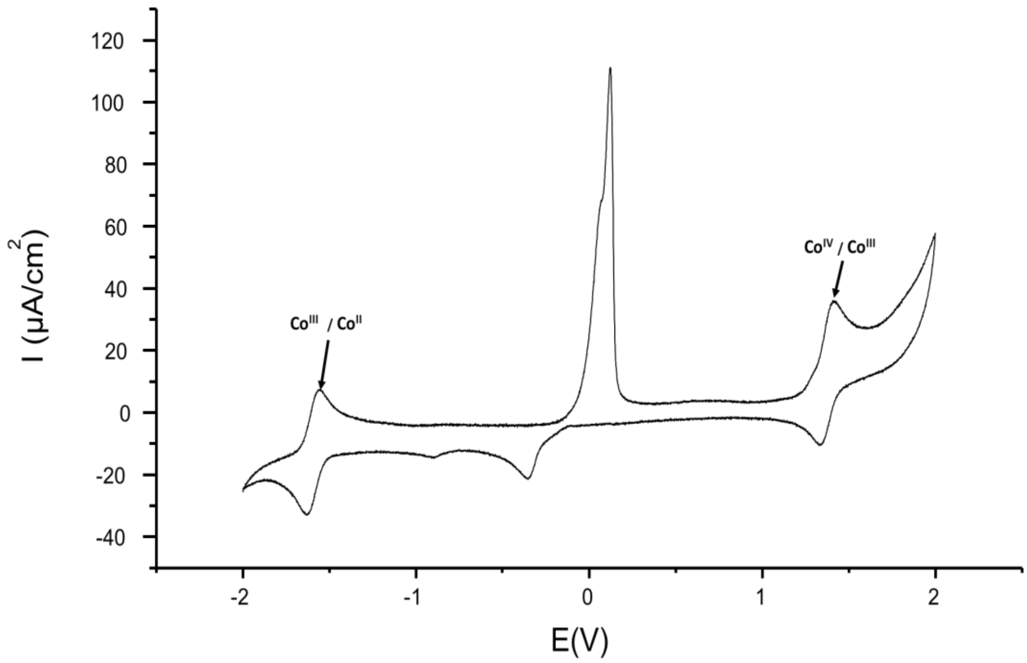

b)

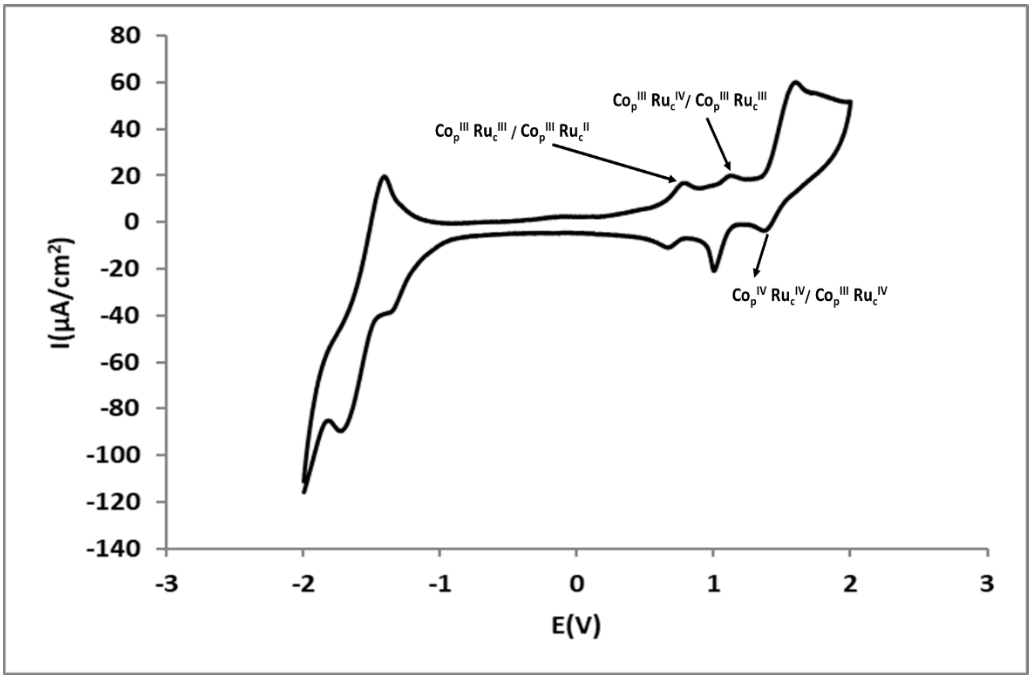

Figure S7. CV for complex **Na[1]** registered in a phosphate buffer (pH= 7.12) vs Ag/AgCl.

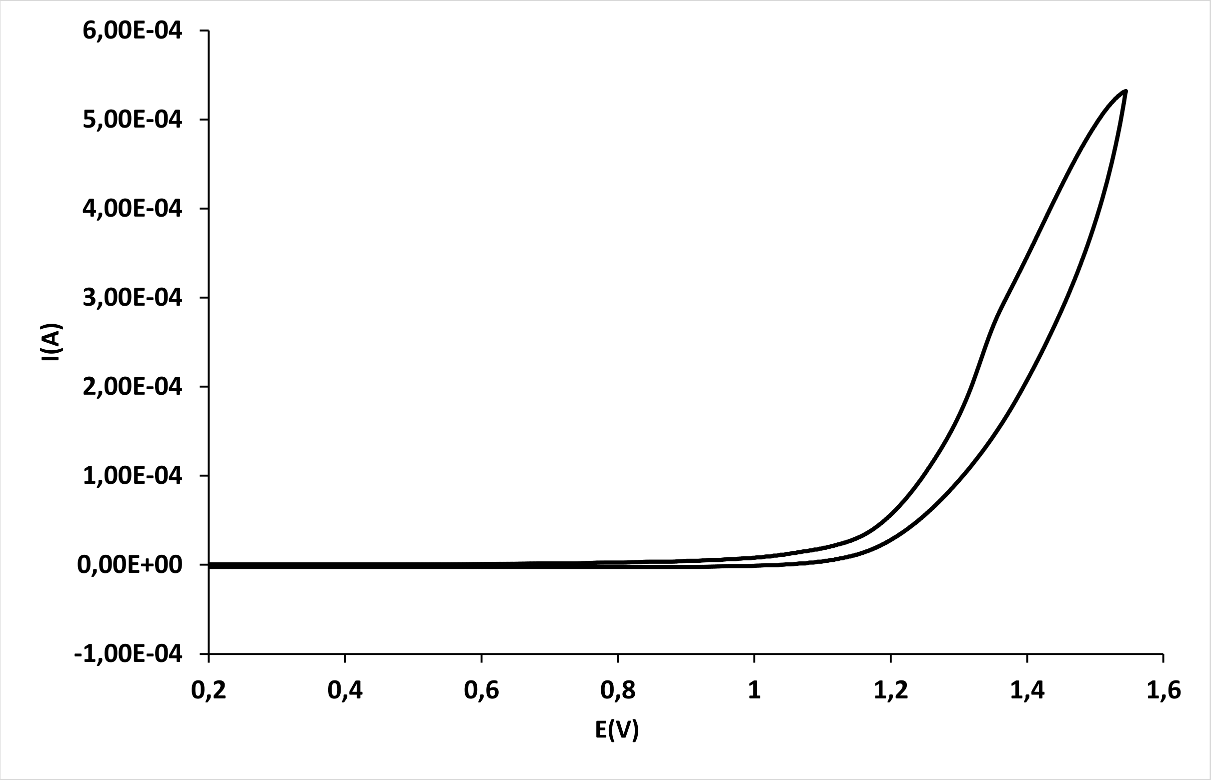

Figure S8. CV for complex **C4** registered in a phosphate buffer (pH= 7.12) vs Ag/AgCl.

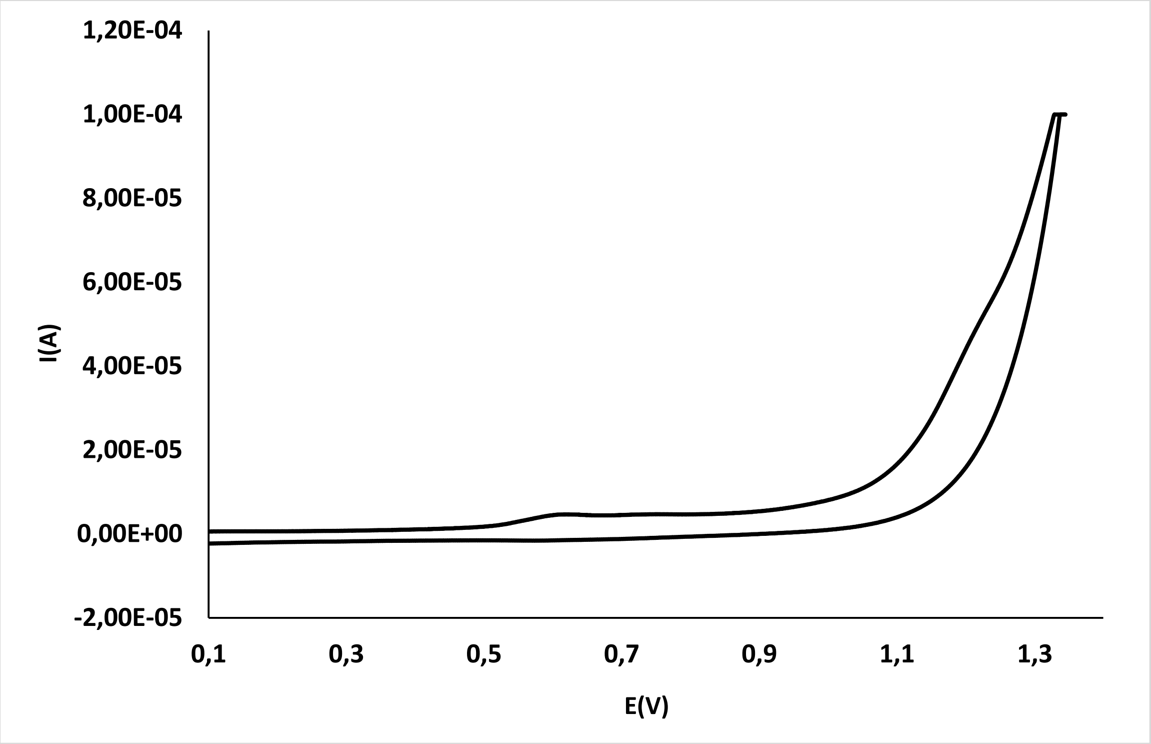

Figure S9. CV of complex **Na[1]** (1 mM) registered in a phosphate buffer (pH= 7.12) vs Ag/AgCl, before and after the addition of 1 mM of a) $CaCl_2$ and b) $ZnCl_2$. The blue and purple inserts show dI/dE to better appreciate the position of the couple Co^{IV}/Co^{III}.

a)

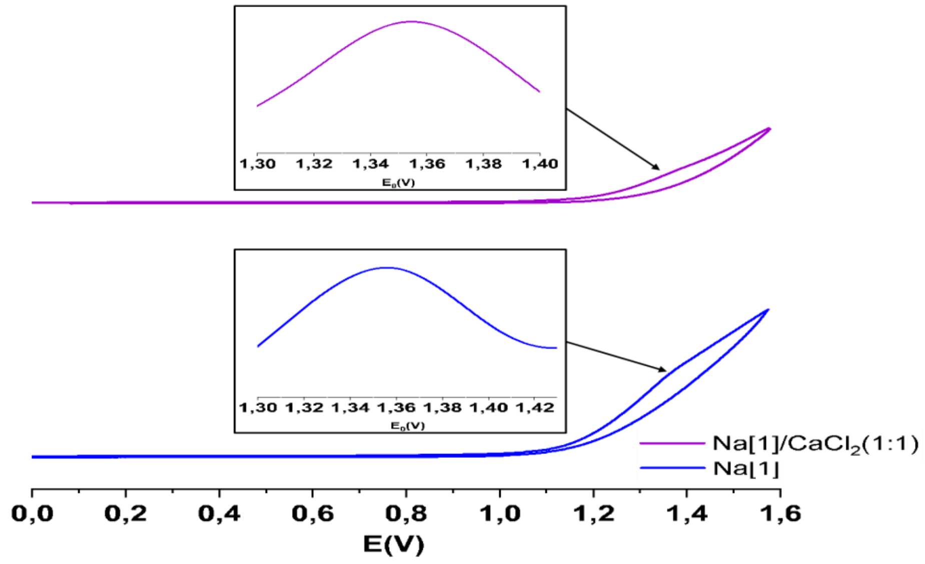

b)

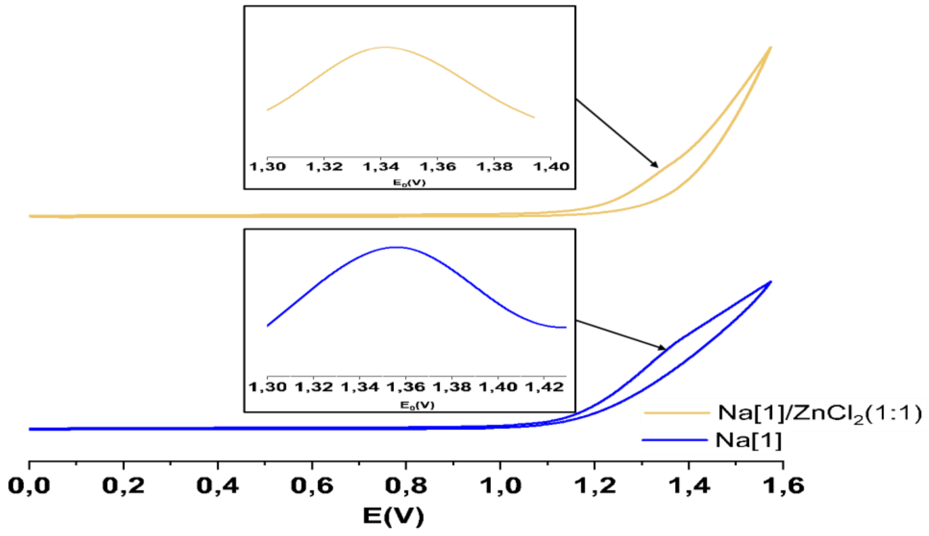

Figure S10. MALDI of **C4** after the photooxidation of 1-phenylethanol. [3,3'-Co(1,2-$C_2B_9H_{11}$)$_2$]$^-$ =324.1 (m/z) and [3,3'Co(1,2-$C_2B_9H_{11}$)(1,2-$C_2B_9H_{11}O$)]$^-$ =340.1 (m/z).

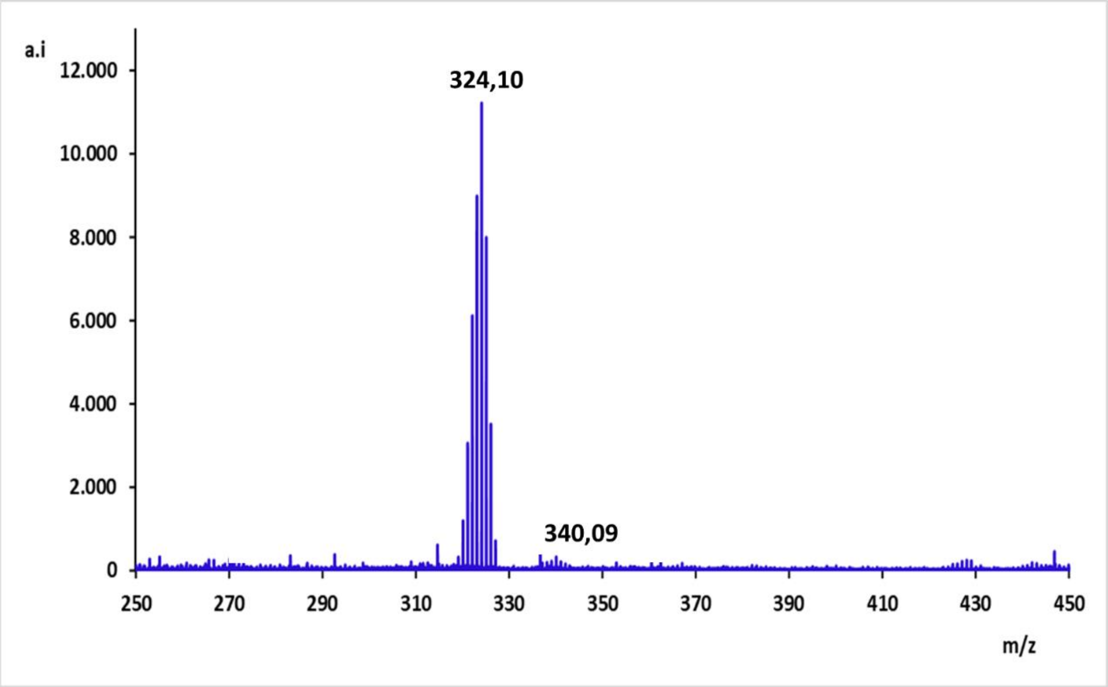

Figure S11. UV-Visible spectra of mixtures of **C2':Na[1]** (1:1) (black line), (1:2) (dotted line) and **C4** (dash dotted line) at a)3 x 10^{-6} and b) 0.33 x 10^{-6} M.

a)

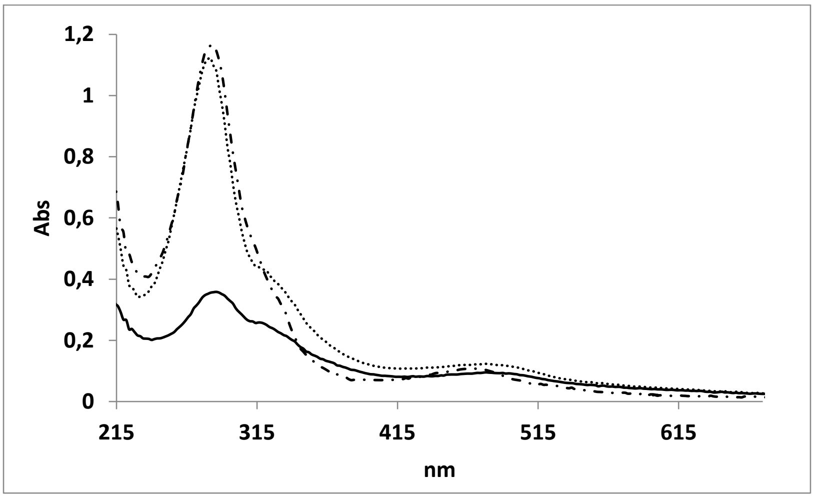

b)

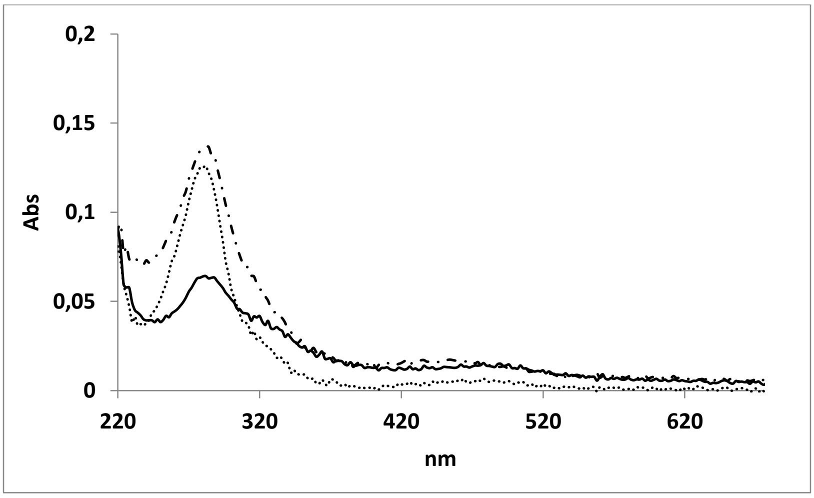

Figure S12. DLS of catalytic mixtures using a) **C4** as catalyst and b) **C2’** and **Na[1]** (1:2) as catalyst.

a)

Sample	Without oxidant ($Na_2S_2O_8$)	With oxidant ($Na_2S_2O_8$)
**S1C4**	D_H = 151nm; PDI = 0.34 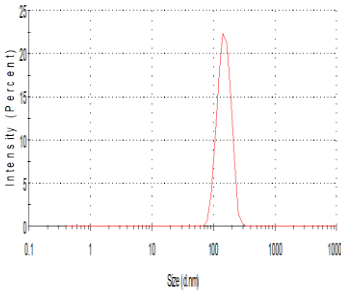 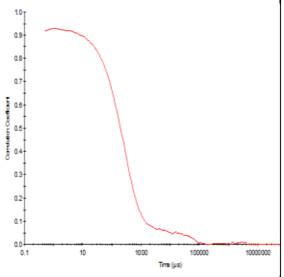	D_H = 140.9nm; PDI = 0.48 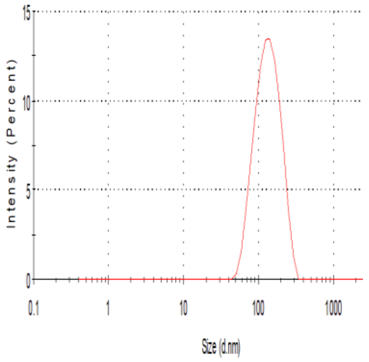 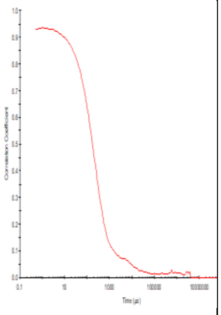

b)

Sample	Without oxidant ($Na_2S_2O_8$)	With oxidant ($Na_2S_2O_8$)
**S2RuCO 1:2**	D_H = 116.1nm; PDI = 0.33 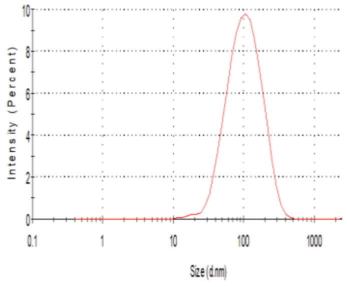 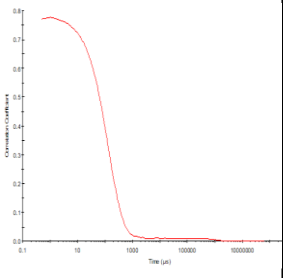	D_H = 128.4nm; PDI = 0.34 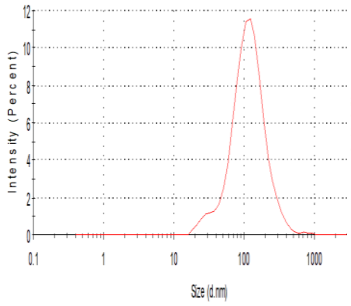 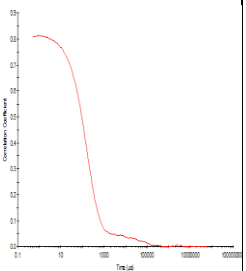

Figure S13. ESI-MS spectra of **C4**.

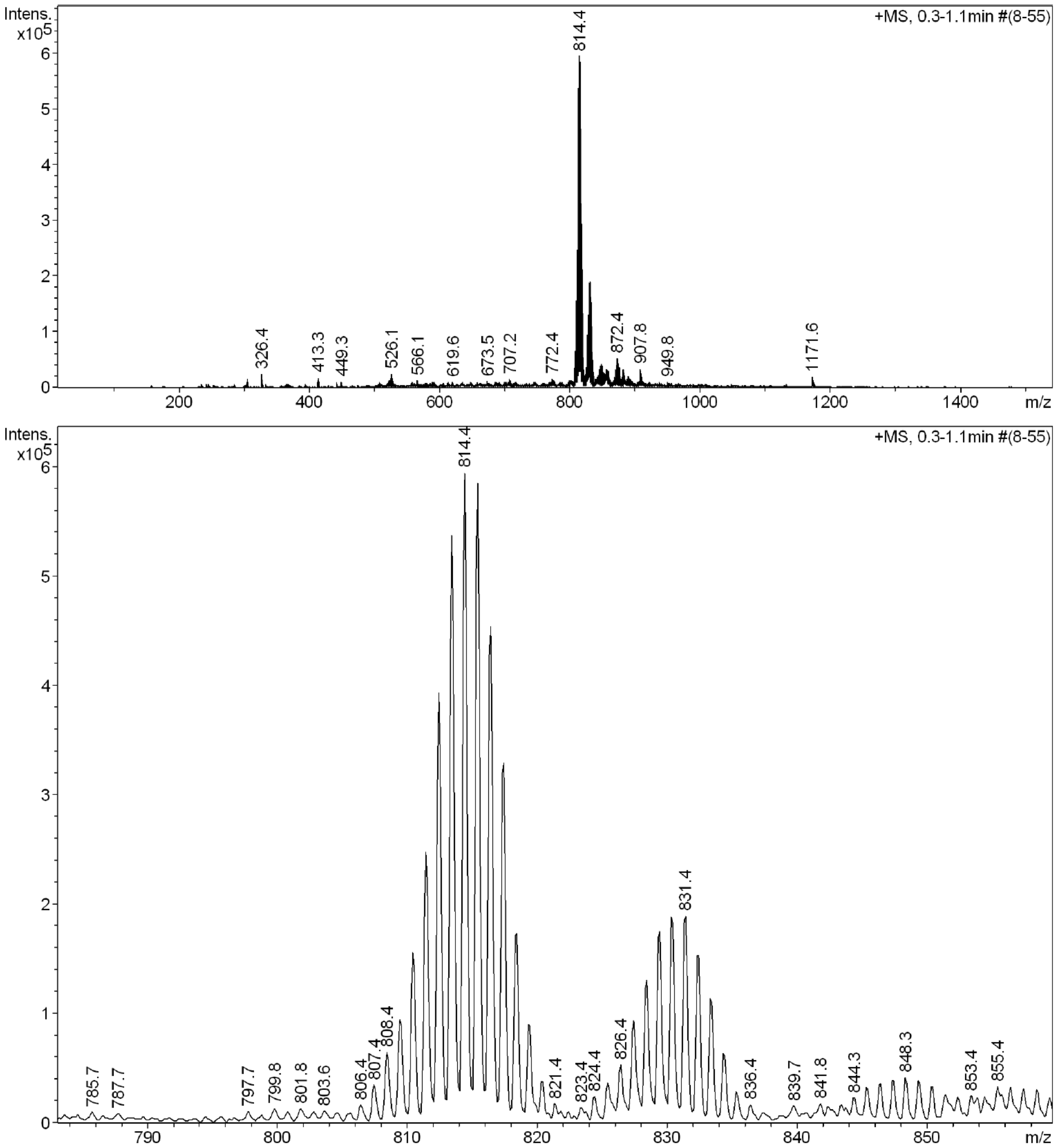

Supplement: Supplementary file 1 — ic1c00751_si_001.pdf [file ic1c00751_si_001.pdf]
